# Natural Product-Inspired Antifungal Drugs Introduced for Human Use from 1872 to August 2026

**DOI:** 10.3390/antibiotics15090938

**Published:** 2026-09-21

**Authors:** Mark S. Butler, Robert J. Capon

**Affiliations:** Institute for Molecular Bioscience, University of Queensland, Brisbane 4072, Australia; r.capon@uq.edu.au

**Keywords:** natural products, antifungal drugs, antifungal resistance, mode of action, drug approval history, β-1,3-D-glucan synthase inhibitors, medicinal chemistry

## Abstract

This review provides a comprehensive account of 30 natural product (NP)-inspired antifungal therapeutic agents introduced for human use between 1872 and August 2026. These comprise 14 NPs (47%) and 16 NP-derived (NP-D; 53%) agents, the latter encompassing semisynthetic derivatives and synthetic analogues of NP scaffolds. These antifungal drugs are organised into eight structural classes, many of which share modes of action (MoA), and are individually classified as NP or NP-D, with accompanying structures illustrating the chemical modifications that link NP-D agents to their progenitor NPs. We summarise the first introduction or approval of each agent for human use by country and year, documented antifungal spectrum, routes of administration, usage status, MoA and key historical, chemical and clinical context. Analysis of the assembled dataset documents the historical importance and enduring clinical impact of these agents, while also revealing a recent concentration of approvals among NP-D inhibitors of β-1,3-D-glucan synthase. These findings underscore the enduring value of NPs as antifungal leads and highlight the need for greater chemical and mechanistic diversity in antifungal discovery, particularly through agents directed at new fungal targets.

## 1. Introduction

Severe fungal diseases are a major, but still under-recognised, contributor to global infectious disease mortality [1,2,3,4]. A 2024 analysis estimated that approximately 6.5 million cases of life-threatening fungal disease, including invasive fungal infections and chronic pulmonary aspergillosis, occur annually, associated with an estimated 3.8 million deaths [1]. This burden is dominated by invasive aspergillosis, invasive candidiasis, *Pneumocystis jirovecii* pneumonia (PJP) and cryptococcal meningitis, for which mortality remains substantial, particularly in severe disease and settings with delayed diagnosis or limited access to effective treatment [1]. By comparison, an estimated 7.7 million deaths in 2019 were associated with bacterial pathogens [5]. Although these global estimates are subject to substantial uncertainty, particularly for undiagnosed and untreated disease, they emphasise a mortality burden comparable in scale with that of other leading infectious diseases and the continuing under-recognition of fungal diseases in surveillance, diagnosis and funding priorities.

The past two decades have also seen escalating antifungal resistance. Multidrug-resistant *Candida auris*, azole-resistant *Aspergillus fumigatus*, terbinafine-resistant *Trichophyton indotineae* and a range of intrinsically resistant moulds have emerged globally, often in settings where therapeutic options are already constrained. Current systemic antifungal classes still have major spectrum gaps, offering unreliable or no activity against challenging moulds such as Mucorales and *Lomentospora prolificans* [6,7]. In parallel, extensive use of agricultural azoles and other fungicides in the environment has contributed to the selection of azole-resistant *A. fumigatus*, with resistant environmental strains now linked to clinical disease, underscoring the need for new antifungal mechanisms and improved stewardship across human, animal and plant health [8]. Human antifungal drugs have also been used to manage superficial and systemic mycoses in companion animals, livestock and other species [9,10,11,12].

While antibacterial drug discovery is widely considered “difficult” [13] antifungal drug development is even more challenging [14,15]. In addition to similar market and resistance constraints, the close evolutionary relationship between fungi and humans results in the conservation of many potential drug targets, limiting selective targeting and increasing the risk of host toxicity [15,16,17,18]. Despite these constraints, a WHO assessment identified 43 antifungal products in clinical or preclinical development as of September 2024 [19].

These clinical and resistance pressures are best understood against the unusual historical trajectory of antifungal therapy. Prior to the 1940s, management of fungal infections largely relied on topical and local treatments such as iodine solutions, mercurial preparations, X-rays, Castellani’s paint (phenol, resorcinol, boric acid and basic fuchsin dye), Whitfield’s ointment (benzoic acid (**3.01**) and salicylic acid (**3.02**), Section 3) [20,21,22], sulfur-salicylic acid ointments, stearic acid (**9.01**) salts (Section 9), Goa (or Araroba) powder (an extract from *Andira araroba* wood that contains chrysarobin) [23,24], croton oil (derived from *Croton tiglium*, which contains phorbol esters) [25,26], gentian violet and malachite green [27]. In the 1940s, two antifungal classes, fatty acids (Section 9) and salicylic acid derivatives (Section 3), entered the market for the treatment of dermatophyte infections. In the 1950s, polyene antifungal drugs (Section 4) provided the first reliable treatments for *Candida*, *Aspergillus* and *Cryptococcus* infections. These three classes are closer in concept to what we now regard as modern antifungal medicines than the earlier antiseptic preparations.

In this review, we describe all natural product (NP) and NP-derived (NP-D) antifungal drugs that have been introduced for human use (Section 3, Section 4, Section 5, Section 6, Section 7 and Section 8), using a framework developed in our earlier review of NP-inspired antibacterial drugs [28]. A companion review, also in *Antibiotics*, describes antifungal agents of synthetic origin and undertakes an overall analysis [29]. The scope, definitions and data conventions used in the present review are described in Section Scope, Definitions and Data Conventions. In addition to the six NP/NP-derived antifungal classes (Section 3, Section 4, Section 5, Section 6, Section 7 and Section 8), the scope of this review has been expanded to include fatty acids (Section 9) and flucytosine (**10.01**) (Section 10), which are more associated with ‘primary metabolism’ [28,30]. Although 8-hydroxyquinoline was reported in 2004 to occur in root exudates of *Centaurea diffusa* [31], the 8-hydroxyquinoline class is classified in this review as synthetic in origin and is covered in the companion review [29]. This classification reflects that 8-hydroxyquinoline, first reported in 1880 [32] and 1881 [33] as a multistep degradation product of cinchonine, was introduced as an antiseptic in Europe around 1895 [34] more than a century before its reported natural occurrence. We also provide a data analysis section with figures outlining historical trends in antifungal development (Section 11), followed by a conclusion assessing the current state of antifungal treatment and possible future directions (Section 12).

## 2. NP Inspired Antifungals

The antifungal agents covered in this review fall into eight distinct antifungal drug classes summarised in Table 1, which details the number of agents introduced for human use in each class, together with their principal modes of action (MoA) and primary targets or processes. Six of the eight antifungal classes (Section 3, Section 4, Section 5, Section 6, Section 7 and Section 8) are conventional NP or NP-D classes, while the two remaining classes are derived from primary metabolites [28], fatty acids (Section 9) and cytosine (**10.02**), the progenitor of flucytosine (**10.01**) (Section 10).

### Scope, Definitions and Data Conventions

Search strategy. Information presented in this review was compiled from peer-reviewed literature, clinical trial registries, biotechnology news sources and the websites of research organisations, companies, government agencies and non-governmental organisations. Relevant sources were identified using drug names, synonyms, trade names and structural-class terms, alone and in combination with the search terms ‘antifungal’, ‘mycosis’, ‘marketed’, ‘introduced’, ‘approved’, ‘clinical use’, ‘mechanism’ and ‘resistance’. Important resources included *The Merck Index* [35], de Haen’s 1976 ‘Compilation of new drugs 1940 thru 1975′ [36], the 2007 edition of the *Pharmaceutical Manufacturing Encyclopedia* [37,38,39,40], the ‘To market, to market’ series of reviews in *Annual Reports in Medicinal Chemistry* [41,42,43] and later *Medicinal Chemistry Reviews* [44,45] and the 2019 analysis of ‘lost medicines’ [46].

In this review, the term NP encompasses directly obtained natural products and clinically marketed NP complexes, whereas NP-D denotes semisynthetic NP derivatives or synthetic agents with a direct and documented structural relationship to a naturally occurring progenitor. The term NP-inspired encompasses NP, NP-D and primary-metabolism-related agents. Although extensive efforts were made to validate the compiled information against authoritative sources, some historical details remain incompletely documented; supporting references are provided throughout.

Therapeutic-entity counting convention. The dataset comprises 30 therapeutic entities (Table 1), each counted once irrespective of formulation, salt form, dosage form, route of administration, trade name, jurisdiction of introduction or subsequent reformulation. Marketed NP or NP-D complexes are counted as single entities, even where they contain multiple major congeners. The total therefore does not correspond to the number of individual compounds depicted in the figures or identified within polyene complexes.

Inclusion criteria for antiseptics. Selected early topical antiseptic, caustic and antifungal agents that predate modern regulatory frameworks have been included only where historical sources document intentional human use for the prevention or treatment of fungal disease. Their inclusion reflects their role in the history of antifungal therapy, rather than implying that all antiseptics with in vitro antifungal activity constitute antifungal drugs.

Introduced versus approved. The term ‘approved’ does not apply to many agents that entered human therapeutic use before modern regulatory systems were established or consistently applied. In the United States, the 1962 Kefauver–Harris Amendments exemplified a major shift towards formal pre-market evaluation of both safety and efficacy [47,48]. The amendments required substantial evidence of effectiveness based on adequate and well-controlled investigations and initiated retrospective assessment of many drugs previously approved primarily on safety grounds. Subsequent regulatory actions also reassessed fixed-dose combinations, including antibiotic–antifungal products, contributing to the withdrawal or restriction of several legacy formulations, including polyene–tetracycline combinations.

Section outlines. A brief commentary is provided for each antifungal agent, addressing, where relevant, its discovering organisation, development history, MoA, resistance mechanisms and other chemical, pharmacological or clinical context, with reference to primary literature where possible. Structures are provided for all antifungal agents, identifying NP and NP-D status and, for NP-D agents, highlighting the functional groups modified relative to the progenitor NPs. Non-quaternary basic compounds are mostly shown as free bases, even when marketed as one or more salts. Each antifungal drug class in Section 3, Section 4, Section 5, Section 6, Section 7, Section 8, Section 9 and Section 10 has associated Tables 2–9, which contain the following information:

- Column 1. A preferred reference name, usually the international nonproprietary name (INN).
- Column 2. A binary descriptor indicating the agent as NP or NP-D.
- Column 3. The earliest substantiated documented event associated with introduction for human antifungal use, including the event type, year and country or countries. A tilde (~) preceding a year indicates that the exact year could not be established and that the reported year is an estimate based on available documentary evidence.
- Column 4. The documented human-use spectrum, ranging from broad-spectrum topical antiseptic activity (Antiseptic) to activity against *Aspergillus* (Asp), *Candida* (Can), *Cryptococcus* (Cry), dermatophytes (Der), *Fusarium* (Fus) and *Malassezia* (Mal). These entries do not imply uniform activity across all species or strains, activity at all anatomical sites or regulatory approval in every jurisdiction.
- Column 5. Routes of administration: aerosol suspension (A), intravenous (IV), oral (O) and topical (T). Antifungal drugs included on the 2025 World Health Organization (WHO) Essential Medicines List [49] or complementary list [49] are denoted as WHO or WHOc, respectively.
- Column 6. Usage status is a best-evidence assessment of commercial availability and human antifungal use at the August 2026 cut-off, rather than a formal regulatory-status classification. Current (C) denotes routine human antifungal use; limited (L) denotes restricted, intermittent or uncertain use or availability; discontinued (D) denotes no identified evidence of continuing routine human antifungal use. Regulatory authorisation alone was not considered evidence of current availability or routine use.

## 3. Benzoic Acid and Salicylic Acid Derivatives

**Figure 1:**
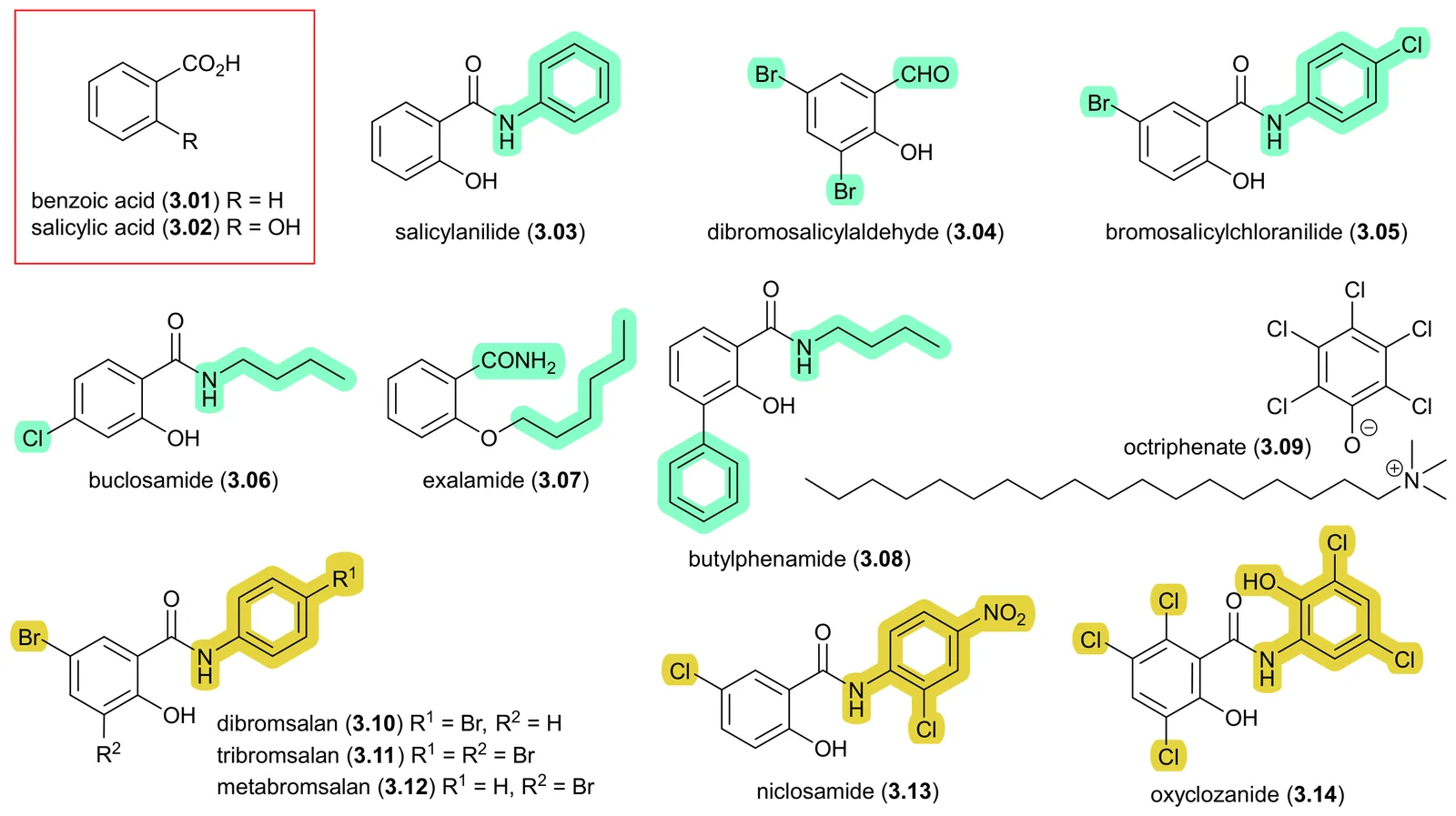
Benzoic acid (**3.01**), salicylic acid (**3.02**) and the salicylic acid-derived antifungals **3.03**–**3.08** introduced into human clinical use, along with octriphenate (**3.09**) used in combination with salicylanilide (**3.03**), NP-D derivatives **3.10**–**3.12** used in antiseptic soap and detergent products and the anthelmintics niclosamide (**3.13**) and oxyclozanide (**3.14**). NPs benzoic acid (**3.01**) and salicylic acid (**3.02**) are highlighted in red; structural variations relative to salicylic acid (**3.02**) are highlighted in green (**3.03**–**3.08**) and mustard (**3.10**–**3.14**).

Benzoic acid (**3.01**) (Figure 1, Table 2), obtained by sublimation of gum benzoin [50], began to be used as an antiseptic around 1872 [51]. Around 1910, combinations of benzoic acid and salicylic acid (**3.02**) [52] such as Whitfield’s ointment were used topically to treat tinea pedis and related dermatomycoses (Section 1) [20,21,22,53]. Arthur Whitfield’s original recipe contained 3% salicylic acid, 5% benzoic acid, soft paraffin and coconut oil [22]. Salicylic acid alone exhibits only limited fungicidal activity; however, its keratolytic action promotes shedding of the stratum corneum, mechanically removing fungi and facilitating the penetration of co-formulated antifungal agents.

**Table 2:** Benzoic acid (**3.01**), salicylic acid (**3.02**) and salicylic acid-derived antifungal drugs introduced into human use.

Name	Origin	Year (Country) First Introduction/Approval [Ref.]	Pathogens	Admin.	Status
Benzoic acid (**3.01**)	NP	~1872 (USA, Europe) [51]	Antiseptic ^1^	T	D
Salicylic acid (**3.02**) + benzoic acid	NP	~1910 (UK) [20]	Der ^2^	T	L
Salicylanilide (**3.03**) ^3^	NP-D	1946 (USA) [36]	Der ^4^	T	D
Dibromosalicylaldehyde (**3.04**)	NP-D	1947 (USA) [36]	Der	T	D
Bromosalicylchloranilide (**3.05**) ^5^	NP-D	~1955 (W. Germany) [54]	Der	T	D
Buclosamide (**3.06**)	NP-D	1958 (W. Germany) [55]	Der	T	D
Butylphenamide (**3.07**)	NP-D	1960 (USA) [36]	Der	T	D
Exalamide (**3.08**)	NP-D	~1980 (Japan) [56]	Der	T	D

^1^ Benzoic acid (**3.01**) is still used as a preservative [57] but not as a stand-alone antiseptic. ^2^ Benzoic acid and salicylic acid (**3.02**) are still used in some topical antifungal and dermatological products. ^3^ Salicylanilide (**3.03**) was also marketed in combination with octriphenate (**3.09**). ^4^ Salicylanilide was also used as a food preservative. ^5^ Marketed as a combination with the antipruritic agent bamipine.

Salicylanilide (**3.03**) was first prepared in 1860 by Schischkoff by heating salicylic acid (**3.02**) with aniline [58]. In 1930, salicylanilide was identified as the preferred antiseptic for protecting textiles against fungal deterioration (Shirlan) [59] and it was subsequently employed as a fungicidal treatment for foods [60]. Salicylanilide, marketed under the trade names Salicylanilide and Salinidol, was introduced in 1946 as a topical treatment for dermatological fungal infections in the USA by several manufacturers [36,61]. However, its clinical use seems to have been relatively short-lived due to its modest activity and the availability of improved treatment options. In 1950, Abbott (USA) introduced Sylamin Cream in the USA, which contained octriphenate (**3.09**) (1.25%, octadecyltrimethylammonium pentachlorophenate) and salicylanilide (5%), for the treatment of tinea capitis [36,62]; however, its use also appears to have been relatively short-lived.

Dibromosalicylaldehyde (**3.04**) (3,5-dibromosalicylaldehyde) was launched onto the USA market in 1947 by Hynson, Westcott & Dunning (USA) as a topical fungicide and antimicrobial (trade name Dalyde) [36]. Dibromosalicylaldehyde was prepared as early as 1900 and used as a precursor in a Perkin reaction [63,64] with acetic anhydride to synthesise 3,5-dibromocoumarin [65]. The antimicrobial properties of halogenated salicylic acid, salicylic aldehyde, and salicylic alcohol derivatives, including dibromosalicylaldehyde, were first systematically characterised in 1937 by Delaunay, who showed that halogenation (particularly dihalogenation and iodination) markedly enhanced activity against *Staphylococcus aureus* [66]. In 1947, Felton and Brewer from Hynson, Westcott & Dunning reported that salicylaldehyde, 5-tert-butylsalicylaldehyde and seven halogenated derivatives had both antifungal and antibacterial activity, with dibromosalicylaldehyde showing the most promising profile [67,68]. Dalyde was available in ointment, solution and powder formulations [69] with some formulations used to treat otitis externa [70]. Beyond this, we identified little further contemporaneous information on Dalyde’s clinical use or marketing history, apart from a notice that Hynson, Westcott & Dunning withdrew the product from the US market in 1984 [71].

Bromosalicylchloranilide (**3.05**) (5-bromo-4′-chlorosalicylanilide, salifungin) is a salicylanilide derivative described in 1954 by Knoll Pharmaceuticals (Germany), with both antidermatophytic and antibacterial activity [72]. Bromosalicylchloranilide showed excellent penetration in a pig skin model and was reported to be essentially non-toxic in acute and chronic animal models [72]. Introduced under the trade name Multifungin around 1955 [54,73], bromosalicylchloranilide was combined with the topical antipruritic bamipine (Soventol) to treat problematic skin and nail infections and relieve itching, erythema and mild allergic skin reactions [54,74,75].

Buclosamide (**3.06**) (*N*-butyl-4-chloro-2-hydroxybenzamide) was launched in September 1958 in West Germany as a topical antifungal by Hoechst AG (Germany) under the trade name Jadit [55]. The product line included the following: (i) Jadit ointment with 2% salicylic acid (**3.02**) and 10% buclosamide; (ii) Jadit solution with 1% salicylic acid and 10% buclosamide; and Jadit H, formulated as (iii) an ointment and (iv) a solution, both with 0.5% hydrocortisone, a mild topical corticosteroid [76,77]. As with other halogenated salicylic acid derivatives, its use declined because of the risk of photo-contact dermatitis [78] and the availability of alternative treatments.

Butylphenamide (**3.07**) (*N*-butyl-3-phenyl salicylamide) is a phenylsalicylamide derivative active against dermatophytes [79,80,81,82] developed by Cutter Laboratories (USA) and introduced in the USA in 1960 under the trade name Bynamid [36]. Lederle Laboratories (USA) were involved with its development [83], but butylphenamide appears to have seen only limited clinical use.

The analgesic and antipyretic properties of exalamide (**3.08**) (2-hexyloxy-benzamide, *o*-hexyloxybenzamide) were reported in 1952 by Herts Pharmaceuticals (UK) [84]. In 1957, T. J. Smith & Nephew (UK) reported that exalamide had promising antifungal activity against dermatophytes [85,86]. In 1974, a patent from the Japanese company S.S. Pharmaceutical Co. (now SSP Co.) described new formulations with improved solubility [87]. From this work, a topical exalamide preparation, Hyperan, started to be used in Japan around 1980 as a Group 2 over-the-counter (OTC) medicine for the treatment of ringworm and athlete’s foot [56,88,89].

Several halogenated salicylanilides, including dibromsalan (**3.10**) (4′,5-dibromosalicylanilide), tribromsalan (**3.11**) (3,4′,5-tribromosalicylanilide) and metabromsalan (**3.12**) (3,5-dibromosalicylanilide), were also used in soap and detergent products for several years [90,91]. However, these halogenated derivatives were gradually replaced by more efficacious antifungals, in part because of concerns about allergic and photoallergic skin reactions associated with their photosensitising properties [74,91,92,93]. Salicylanilides have also been used as anthelmintic drugs such as niclosamide (**3.13**), introduced by Bayer (Germany) in 1959 and widely used in human and veterinary medicine [94,95]. Recently, interest has emerged in repurposing niclosamide as an antifungal [95,96] and oxyclozanide (**3.14**), another veterinary anthelmintic, as an antibacterial [97].

Salicylic acid derivatives MoA. The antifungal activities of salicylanilide (**3.03**) and bromosalicylchloranilide (**3.05**) were characterised principally through phenotypic antimicrobial and antidermatophytic studies, and a drug-specific molecular target or antifungal MoA has not been established for either agent [67,68,72,98]. More recent mechanistic studies have instead focused on the structurally related halogenated salicylanilide anthelmintics niclosamide (**3.13**) [95] and oxyclozanide (**3.14**) [96] during drug repurposing efforts. These studies show that halogenated salicylanilides can behave as protonophores, disrupt mitochondrial membrane potential and oxidative phosphorylation, and induce retrograde signalling and broad metabolic stress in *Candida albicans* and other yeasts [95,96,99]. At modest fungistatic concentrations, niclosamide and/or oxyclozanide also inhibit filamentation, biofilm formation and tissue invasion, consistent with an antivirulence-dominated phenotype [96,99,100]. In bacteria, bromosalicylchloranilide has also been associated with membrane perturbation and disruption of energy and ion homeostasis [75]. Although their shared halogenated salicylanilide scaffold provides a plausible basis for extrapolating these findings to related salicylanilide derivatives, direct mechanistic studies in relevant fungal systems are required to establish the extent to which this mechanism is shared across the class.

Although no drug-specific fungal target or antifungal MoA has been established for dibromosalicylaldehyde (**3.04**), studies of related salicylaldehydes suggest that the *o*-hydroxyaldehyde motif contributes to antimicrobial activity [101,102,103,104,105]. In related salicylaldehydes, halogenation has been proposed to enhance fungal and bacterial membrane penetration and accumulation, while modulating the electronic properties and metal-chelating behaviour of the salicylaldehyde core. Aldehydes can also form Schiff bases with amine-containing proteins, peptides and small molecules [106,107,108]. Therefore, the antimicrobial activity of halogenated salicylaldehydes may reflect a combination of membrane perturbation and multiple covalent and metal-mediated interactions, rather than a single defined enzyme target.

Salicylic acid derivatives resistance. Salicylanilides were largely confined to topical dermatological use and, later, to anthelmintic and veterinary indications. Although no well-defined patterns of clinical resistance to salicylanilides have been reported, halogenated salicylanilides have been shown to retain in vitro activity against *C. albicans* clinical isolates with common azole- and echinocandin-resistance mechanisms [95,96,99]. Susceptibility to salicylanilide derivatives nevertheless varies substantially among fungal species and strains; studies of synthetic analogues found generally greater activity against moulds than yeasts, with often limited activity against *Candida* spp. [109].

## 4. Polyenes

**Figure 2:**
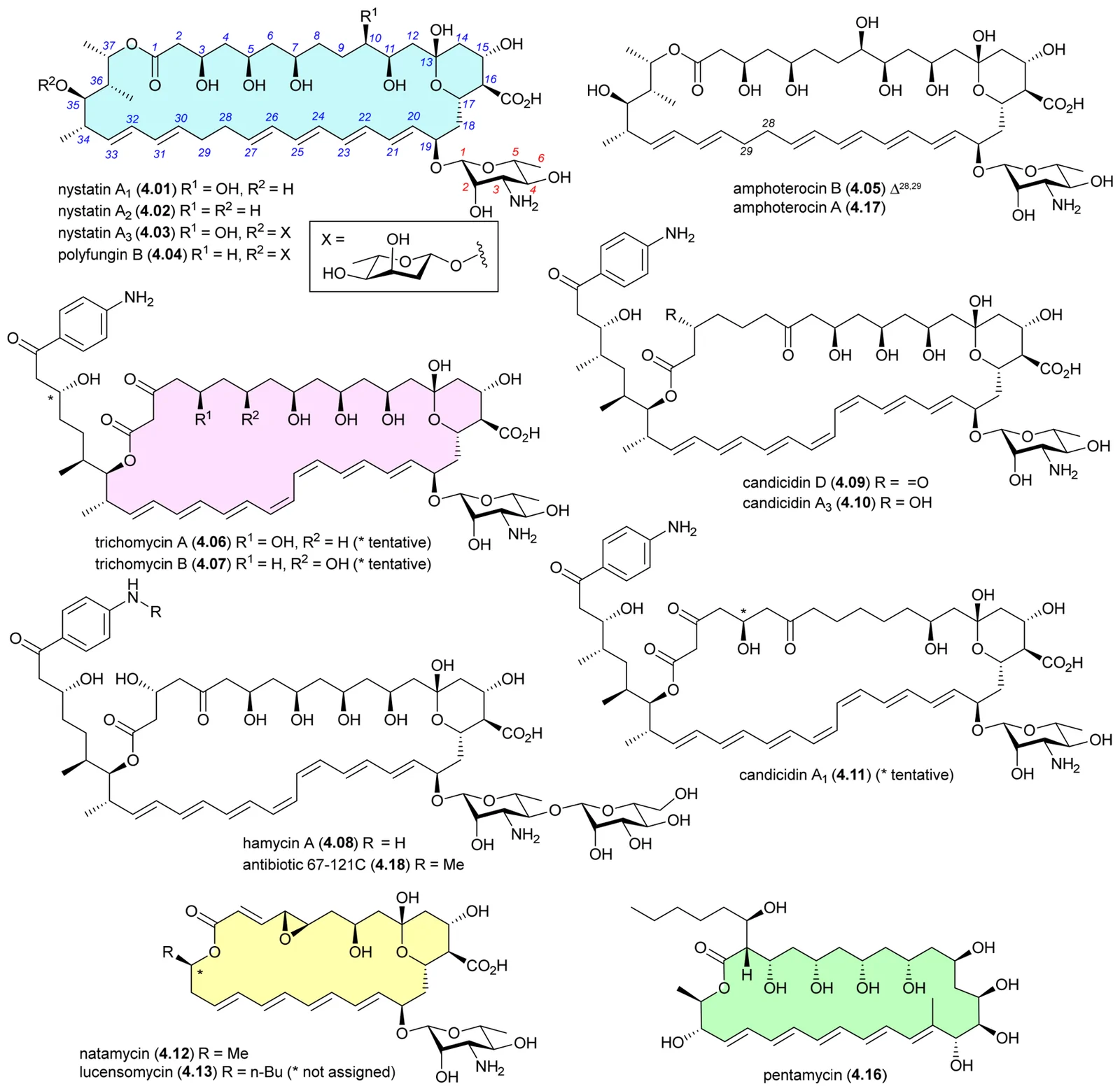
NP polyenes **4.01**–**4.13** and **4.16** introduced or approved as antifungal drugs or complex components, along with antibiotic 67-121C (**4.18**). Highlighting: Subclasses I (blue), II (pink), III (yellow) and IV (green) based on macrolactone structure diversity.

**Figure 3:**
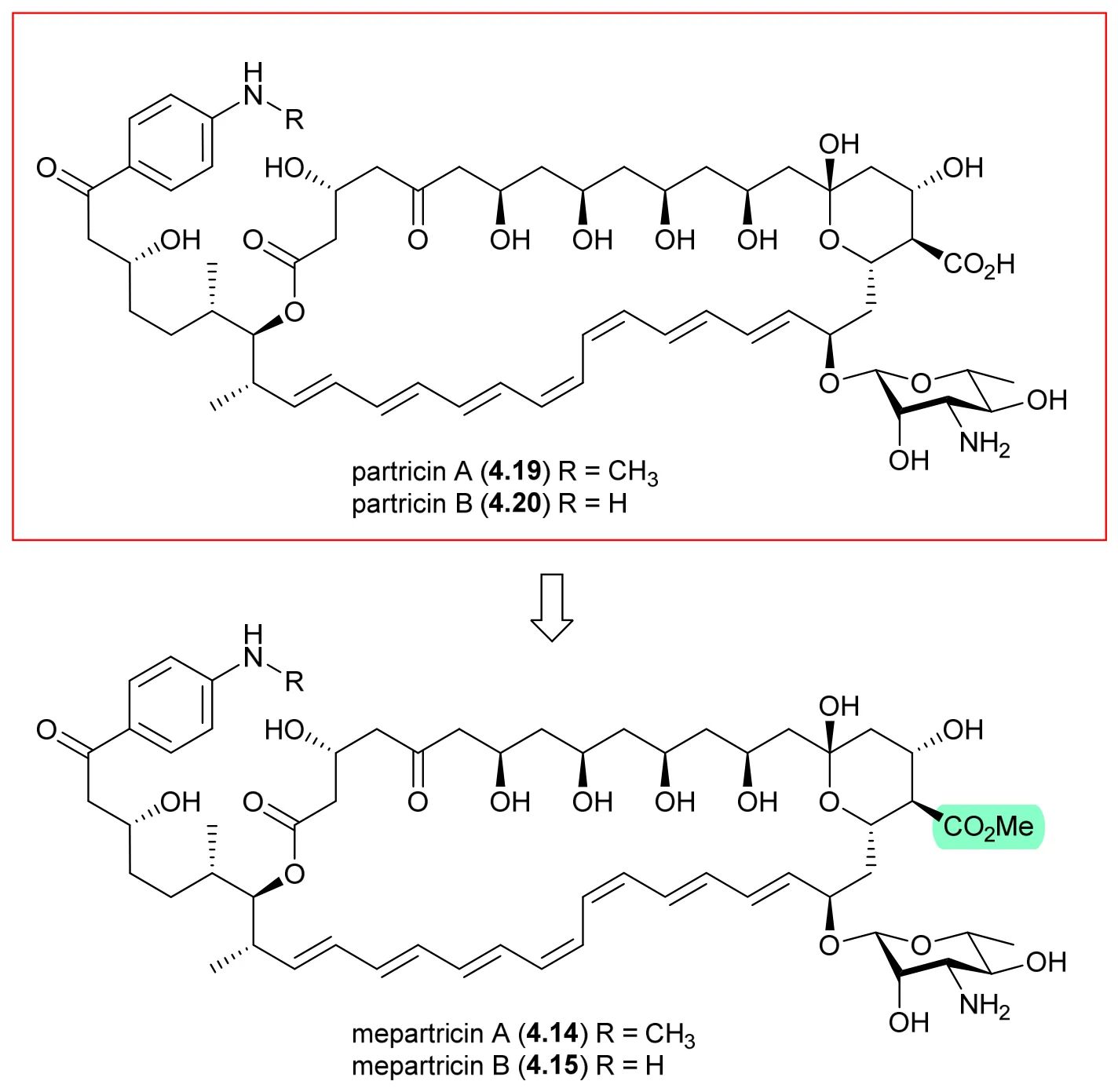
The NP-D complex major components mepartricin A (**4.14**) and B (**4.15**) introduced for human use as antifungal drugs, derived from the partricin complex major components partricin A (**4.19**) and B (**4.20**). The NP partricins are highlighted in a red box, and the structural variations in mepartricins are highlighted in green.

The polyenes (Figure 2 and Figure 3) are a class of strongly antifungal polyketide macrolactones that contain multiple conjugated double bonds; most are glycosylated with the aminosugar mycosamine. They are among the earliest antifungal drugs, with nine polyene drugs or complexes introduced for human use (Table 3). These can be divided into four structural subclasses (Figure 2), highlighted in blue, pink, yellow and green. While the extended conjugated polyene systems common to these subclasses confer potent antifungal activity, they also confer chemical lability, with susceptibility to photoisomerisation, oxidative degradation, acid- and base-catalysed degradation and aggregation in aqueous solution [110,111,112,113].

**Table 3:** Polyene antifungal drugs introduced or approved for human use.

Name	Origin	Year (Country) First Introduction/Approval [Ref.]	Pathogens/ WHO	Admin.	Status
Nystatin complex (**4.01**–**4.04**) ^1^	NP	1954 (USA) [36]	Can/WHO ^2^	O, T	C
Amphotericin B (**4.05**)	NP	1958 (USA) [36]	Can, Cry, Asp/WHO ^3^	IV	C
Hachimycin complex (**4.06**, **4.07**) ^4^	NP	~1958 (Japan) [114]	Can, Der	T, IV	D
Hamycin complex (**4.08**) ^5^	NP	1963 (India) [115]	Can, Der	O, T	D
Candicidin complex (**4.09**–**4.11**) ^6^	NP	1964 (USA) [36]	Can, Der	T	D
Natamycin (**4.12**)	NP	~1964 (UK) [116]	Asp, Can, Fus ^7^	T, A	C
Lucensomycin (**4.13**)	NP	~1964 (Italy) [117,118,119]	Can, Der	T	D
Mepartricin complex (**4.14**, **4.15**) ^8^	NP-D	~1977 (Italy) [120]	Can, Der ^9^	T	D
Pentamycin (**4.16**)	NP	1980 (Switzerland) [121,122]	Can, Der	T	D

^1^ Marketed as a complex inclusive of nystatin A_1_ (**4.01**), A_2_ (**4.02**) and A_3_ (**4.03**), as the major components; Chinese nystatin contains nystatin A_1_, A_3_ and polyfungin B (**4.04**) as the major components. ^2^ Nystatin is also used in animal health. ^3^ Amphotericin B (**4.05**) is also used to treat visceral leishmaniasis and in animal health. ^4^ Marketed as a complex inclusive of trichomycin A (**4.06**) and B (**4.07**). ^5^ Marketed as a complex inclusive of hamycin A (**4.08**). ^6^ Marketed as a complex inclusive of candicidin D (**4.09**), A_1_ (**4.10**) and A_3_ (**4.11**). ^7^ Natamycin (**4.12**) is also used in animal health and as a food preservative. ^8^ Semi-synthetic complex inclusive of mepartricin A (**4.14**) and B (**4.15**), also sold in combination with tetracycline (Tricangine). ^9^ Mepartricin was later used as an orally administered treatment for benign prostatic hyperplasia (BPH) and chronic non-bacterial prostatitis/chronic pelvic pain syndrome (CPPS).

Polyene Sub-class I. The nystatin complex components **4.01**–**4.04** and amphotericin B (**4.05**) are 38-membered macrolactones bearing a mycosamine sugar that differ slightly in hydroxylation and share all-*E*-configured polyene subunits. Initially termed ‘fungicidin’, and later renamed nystatin (after New York State) [123], the nystatin complex was isolated from *Streptomyces noursei*, which was recovered from soil collected in Fauquier County (VA, USA) by Rachel Fuller Brown and Elizabeth Lee Hazen [124,125] at the New York State Department of Health. First disclosed in a 1950 conference abstract [126], with a full paper following in 1951 [127], the nystatin complex comprises several closely related polyene macrolides, including nystatin A_1_ (**4.01**), A_2_ (**4.02**) [128,129] and A_3_ (**4.03**) [130,131]. Of note, a Chinese version of nystatin complex (Nysfungin) comprises nystatin A_1_ and A_3_, and polyfungin B (**4.04**) [132,133,134]. The planar structure of nystatin A_1_ was elucidated in 1970 as a 38-membered macrolactone bearing a mycosamine sugar [135], with its absolute configuration determined in 1989 [136]. Its biosynthetic pathway has since been characterised in detail [129,137,138]. Nystatin A_3_ and polyfungin B both have an α-L-digitoxose at C-35 [133,139].

Marketed as Mycostatin, nystatin was introduced in the USA by E. R. Squibb & Sons (USA) for the treatment of vaginal candidiasis [36]. As the first polyene and one of the first antifungal drugs, nystatin represented a major advance in antifungal therapy, although its clinical use was initially restricted to topical and oral non-absorbed indications because it was not systemically absorbed after oral administration, whereas parenteral use was limited by toxicity concerns [140]. Nystatin is still widely used around the world [134,140,141] and is on the WHO’s Essential Medicines List as a lozenge (mild oropharyngeal candidiasis), pessary (vulvovaginal candidiasis) and liquid (oropharyngeal candidiasis) or solid (gastrointestinal candidiasis) oral dosage forms [49]. It is also available as topical cream, ointment and powder preparations to treat skin fungal infections [142]. However, nystatin was unsuitable as an orally or parenterally administered agent for systemic mycoses, as it has negligible intestinal absorption and IV administration was associated with severe local venous irritation [143]. Beyond its clinical legacy, the two discoverers established the Brown–Hazen Research Fund and assigned their patent royalties—totalling over USD 13 million—to the non-profit Research Corporation for Science Advancement [124,125]. This funding supported medical-mycology research until the late 1970s, after which the remaining funds were used to establish perpetual scholarships for women scientists [125].

Fixed-dose combinations of antibacterial and antifungal agents, predominantly nystatin (**4.01**) products with and without corticosteroids, saw widespread use in the USA from 1955 through the 1970s and remained available in some other countries until at least the 1990s [144]. For example, a combination of nystatin (**4.01**) and tetracycline was launched by E. R. Squibb & Sons (USA) as Mysteclin (later renamed Mysteclin-V) in the USA in 1955 [145]. Mysteclin, Lederstatin (nystatin and demeclocycline, 1967), Siltetrin (nystatin and tetracycline, 1971) and Bristrex (nystatin and tetracycline) were all sold in the UK at the start of 1972 [146]. Squibb also sold an amphotericin B (**4.05**) and tetracycline cream called Mysteclin-F from around 1960 [147]. Other antifungal combinations identified were Bristol-Myers Squibb’s Triadcortyl (nystatin, neomycin, gramicidin and triamcinolone acetonide), Bayer’s Nystaform (nystatin and the 8-hydroxyquinoline iodochlorhydroxyquin) and Nystaform-HC (nystatin, iodochlorhydroxyquin and hydrocortisone), and nystatin and triamcinolone acetonide (various manufacturers). In the late 1960s, the US FDA systematically re-evaluated legacy medicines, particularly antibiotic combinations, under the post-Kefauver–Harris Amendments requirement for ‘substantial evidence’ of efficacy [148,149]. A review by the National Research Council concluded that there was insufficient evidence to support the effectiveness of Mysteclin combinations for their labelled indications, which focused on preventing or treating *Candida* overgrowth during tetracycline therapy. Contemporary commentary also criticised fixed-dose combinations for promoting empirical use and limiting dose flexibility relative to individual patient needs. Accordingly, the FDA moved to remove such antibacterial-antifungal combinations from the list of certifiable drugs and to withdraw their approvals; this action was upheld following a legal challenge by Squibb [150].

The potent antimycotic amphotericin complex was first reported in 1956 by the Squibb Institute for Medical Research (USA) from *Streptomyces nodosus* [151], isolated from soil collected in the Orinoco River region of Venezuela [152,153,154,155]. The complex comprised predominantly amphotericin B (**4.05**), together with approximately 10% of the minor congener amphotericin A (**4.17**). Although the structure of the mycosamine sugar present in both the nystatin and amphotericin complexes was elucidated in 1957 [156], the complete structure and absolute configuration of amphotericin B were determined by X-ray crystallography in 1970 [157]. In 1985, amphotericin A was confirmed to be a 28,29-dihydro analogue of amphotericin B, featuring the same diene–ethane–tetraene chromophore present in the nystatin components **4.01**–**4.04** [158]. Its absolute configuration was established in 1996 [159]; however, its antifungal and physicochemical properties were too similar to nystatin to warrant further development as a separate drug. Conversely, amphotericin B displayed more potent antifungal activity and was active in animal models of systemic mycoses [143]. However, amphotericin B is not systemically absorbed after oral administration in humans. This limitation was addressed by formulation with sodium deoxycholate, which produced a lyophilisable complex that, upon reconstitution in 5% glucose, formed a micellar suspension suitable for IV infusion. This IV deoxycholate formulation of amphotericin B was developed by E. R. Squibb & Sons (USA) and approved in the US in 1958 under the trade name Fungizone [36] as a treatment for candidiasis and cryptococcal meningitis [160]. Amphotericin B has broad-spectrum antifungal activity against most yeasts (e.g., *Candida*, *Cryptococcus*), dimorphic fungi (e.g., *Histoplasma*, *Blastomyces*, *Coccidioides*), *Aspergillus* spp. and members of the order Mucorales [161]. Resistance remains rare in susceptible fungi, although reduced susceptibility is observed in some *Candida* spp., *Fusarium* spp. and *Aspergillus terreus* [162,163,164,165]. Despite its well-recognised nephrotoxicity and infusion-related reactions, amphotericin B has remained a cornerstone of antifungal therapy for decades, particularly as the number of immunocompromised patients increased in the 1980s [166,167].

The next advance was the approval of three new lipid-based amphotericin B formulations in the 1990s, which substantially improved tolerability while preserving antifungal efficacy. A liposomal amphotericin B (L-AMB) formulation, developed by Vestar (USA), received marketing authorisation in Ireland in December 1990 and was approved in the USA in August 1997 as AmBisome. Next, an amphotericin B colloidal dispersion (ABCD), developed by Liposome Technology (USA), was licensed in the UK in October 1994 as Amphocil and approved in the USA in August 1996 as Amphotec. Finally, a lipid complex formulation (ABLC) developed by The Liposome Company (USA) was approved in November 1995 in the USA and subsequently authorised in several European countries from 1996 as Abelcet. AmBisome (L-AMB) [168] remains the most widely used lipid-based formulation [169], whereas Abelcet [170] is used less frequently, principally owing to cost, availability and local practices [171]. Clinical use of the ABCD formulation (Amphocil/Amphotec) has effectively been discontinued because of higher rates of infusion-related reactions, and manufacture ceased in 2011 [172]. Both the sodium deoxycholate and L-AMB formulations are listed on the 2025 WHO Essential Medicines List; L-AMB has a better safety profile and should be prioritised where local availability and cost permit [49]. Amphotericin B is also used to treat visceral leishmaniasis, a sandfly-borne parasitic disease that predominantly affects people in parts of Latin America, East Africa and the Indian subcontinent [173,174,175].

Polyene Sub-class II. The NP hachimycin (**4.06** and **4.07**), hamycin (**4.08**) and candicidin (**4.09**–**4.11**) complexes, and NP-D mepartricin complex (**4.14** and **4.15**) derived from partricin complex (**4.19** and **4.20**), share 38-membered macrolactones and mycosamine subunits, a *para*-amino phenyl ketone-containing side chain, and a conserved all-*E*,*E*,*Z*,*Z*,*E*,*E*,*E*-heptaene chromophore; they differ slightly in hydroxylation and ketone substitution patterns. Hamycin (**4.08**) also has a D-mannosyl-D-mycosaminyl disaccharide.

The hachimycin (trichomycin) complex was first reported in 1952 from *Streptomyces hachijoensis*, which was isolated from a soil sample collected on the volcanic island Hachijō-jima, approximately 287 km south of Tokyo [176]. Hachimycin exhibited trichomonacidal and antifungal activity and was developed by Fujisawa (Japan) for clinical use around 1958 [114]. It remains listed on the 18th Edition of the Japanese Pharmacopoeia (2021) [177], although no currently marketed product was identified. The planar structures of two major complex components, trichomycin A (**4.06**) and B (**4.07**), were described in 1990 [178,179]. In 2025, the structure of trichomycin A was revised, and the absolute configurations of trichomycins A and B were assigned [180].

The hamycin complex [181,182,183,184] was first reported from *Streptomyces pimprina*, isolated from soil collected in Pimpri, Poona, India, in 1961 [185,186]. Although no peer-reviewed structure elucidation studies have been published, hamycin A (**4.08**) has been reported to be identical to antibiotic 67-121B, a demethyl analogue of 67-121C (**4.18**) [187]. The structure of 67-121C was determined to contain a D-mannosyl-D-mycosaminyl disaccharide, and insights into its absolute configuration were obtained by analysis of its biosynthetic gene cluster [131,188,189,190]. Hamycin was marketed by Hindustan Antibiotics (India) from 1963 as a topical ointment and pessaries primarily to treat *Candida* and other yeast or dermatophyte infections [191] and was also available as a suspension to treat oral thrush and vaginal infections; however, its clinical use appears to have been discontinued.

The candicidin complex was first reported in 1953 from *Streptomyces griseus*, isolated from a sample of cow manure collected in New Jersey (USA) [192,193,194]. The candicidin complex consists of three major components: candicidin D (**4.09**) (levorin A_2_, ascosin A_2_) [195,196], A_1_ (**4.10**) (levorin A_1_, ascosin A_1_) [187,197,198] and A_3_ (**4.11**) (levorin A_3_, ascosin A_3_) [199], for which stereochemical assignments were reported in 2015, 2015 and 2020, respectively. In 1964, Julius Schmid Pharmaceuticals (USA) introduced candicidin (Candeptin, generic Vanobid) onto the US market [36] to treat candidiasis as a dermatological ointment and intravaginal preparation for vulvovaginal candidiasis. Although candicidin was also used in other parts of the world, by the 1980s it had largely fallen out of routine clinical use, as topical and oral azoles and, in selected cases, nystatin became preferred for vulvovaginal candidiasis.

Mepartricin (SPA-S-160; methyl partricin) comprises two principal components, mepartricin A (**4.14**) and mepartricin B (**4.15**) (Figure 3) [200], which are methyl ester derivatives of the partricin complex components partricin A (**4.19**) and partricin B (**4.20**) [201]. This complex was isolated from *Streptomyces aureofaciens* [201], an actinomycete recovered from soil collected in central Italy [202]. Developed by Società Prodotti Antibiotici (SPA, Italy), mepartricin was initially introduced around 1977 as an antifungal agent for the treatment of vaginal infections caused by *Candida* spp. and the protozoan *Trichomonas* spp., and was marketed under the trade names including Tricandil and Ipertrofan [120]. A vaginal cream formulation (Tricangine) containing mepartricin combined with tetracycline has also been used for vulvovaginitis caused by *T. vaginalis*, *C. albicans* and mixed bacterial infections. Subsequently, mepartricin was repurposed for the treatment of benign prostatic hyperplasia (BPH) [203] and chronic non-bacterial prostatitis/chronic pelvic pain syndrome (CPPS) [204]. Mepartricin’s therapeutic rationale is based on its ability to bind sex hormones, including 17β-oestradiol, testosterone and dihydrotestosterone, and thereby reduce their intestinal reabsorption and modulate their bioavailability [205]. This is proposed to reduce circulating sex-hormone concentrations and limit prostatic enlargement in BPH without major systemic endocrine effects.

Polyene Sub-class III. Natamycin (**4.12**) and lucensomycin (**4.13**) share a 26-membered macrolactone system, a mycosamine glycoside, an *E*-configured α,β-unsaturated bond and an all-*E*-configured tetraene; they differ only in the length of the polyketide side chain.

Natamycin (**4.12**) (pimaricin, tennecetin, antibiotic A 5283) is a tetraene first reported in 1957 by Gist-Brocades Research Laboratories (Netherlands) from *Streptomyces natalensis*, which was isolated from soil collected near Pietermaritzburg, South Africa [206,207]. Natamycin has also been isolated from other *Streptomyces* species [208,209]. The non-hemiketal planar structure of natamycin was determined in 1966 [210]. The hemiketal moiety was confirmed in 1976 [211] and absolute configuration was established in 1990 [212]. Natamycin may have been introduced in Belgium in 1963 as Pimafucin by Mycopharm, a pharmaceutical division of Gist-Brocades; however, contemporaneous regulatory, product or other documentary evidence substantiating this introduction was not identified. The earliest substantiated regulatory event located was Mycopharm’s UK approval in December 1964 for an aerosol suspension of Pimafucin for the treatment of pulmonary aspergillosis [116]. In 1969, UK approval was granted for a topical cream and vaginal tablets [213], while a suspension formulation was approved to treat oral thrush in 1974 [214,215]. Natamycin was approved by the US FDA in 1978 for topical ophthalmic treatment of fungal blepharitis, conjunctivitis and keratitis caused by susceptible fungi, including *Fusarium solani* keratitis (trade name Natacyn) [215,216,217,218]. Natamycin is widely used in the food industry to inhibit fungal growth on the surfaces of foods such as cheeses and sausages, helping to prevent fungal spoilage and associated mycotoxin production [219,220]. Natamycin has also been used in animal health, particularly to treat ringworm infections in cattle and horses [10].

Lucensomycin (**4.13**) (etruscomycin, lucimycin, antibiotic FI 1163) was first reported in 1957 from *Streptomyces lucensis*, which was isolated from soil collected near Lucca, Tuscany, Italy [119,221,222]. ‘Etruscomicina’, an ointment containing lucensomycin developed by Farmitalia (Italy), was introduced around 1964 to treat dermatoses and *Candida* infections [117,118,119]; however, it is no longer sold. The structure of lucensomycin, which is closely related to natamycin (**4.12**), was proposed [223,224] in 1966 and shown to exist as a cyclic hemiketal in 1972 [211,225]. Although the mycosamine stereochemistry was determined in 1978 [126], the absolute configuration of the aglycone has not currently been reported. Despite this, lucensomycin has generally been depicted with the same absolute configuration as natamycin, including in semi-synthetic studies that used lucensomycin directly sourced from Farmitalia [226,227,228]. The biosynthetic gene cluster of lucensomycin has been investigated [229,230,231], and the proposed structure was supported by NMR analysis [229,232].

**Figure 4:**
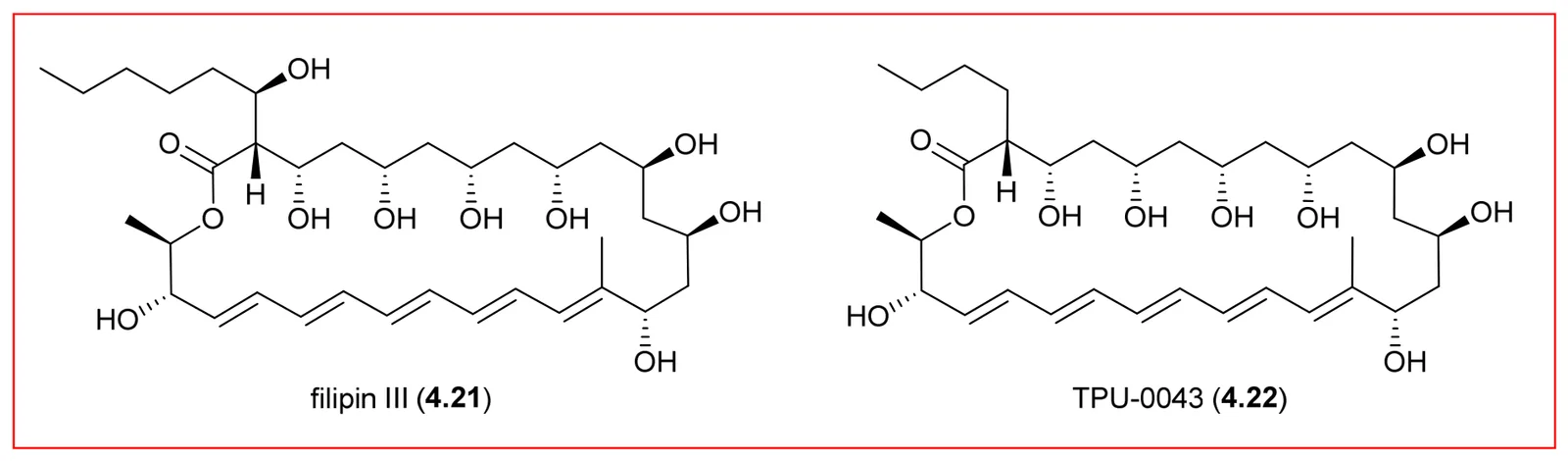
NPs filipin III (**4.21**) and TPU-0043 (**4.22**), structurally related to pentamycin (**4.16**).

Polyene Sub-class IV. Pentamycin (**4.16**) (fungichromin, lagosin, antibiotic A246, cogomycin, 14-hydroxyfilipin III) is a polyene antifungal first reported in 1955 from *Streptomyces cellulosae*, obtained from soil collected near Greenville (Texas, USA) [233,234]. It features a 26-membered macrolactone with an all-*E*-configured pentaene, lacking both glycosylation and the acetal heterocycle common to other approved polyene antifungals, and with a hexyl branch at C-2. The planar structure for **4.16** was first reported in 1960 as lagosin [235,236] and in 1962 as fungichromin [237], with the two structures confirmed to be identical by NMR in 1982 [238,239]. In 1995, the absolute configuration of the closely related filipin III (**4.21**, Figure 4), isolated from *Streptomyces filipinensis* [240], was determined using NMR analysis of three acetonide derivatives and application of the advanced Mosher’s ester method [241]. The relative configuration of pentamycin was determined in 1989 using acetonide and degradation studies [242] and its absolute configuration was established in 2023 using total synthesis [243]. An X-ray crystal structure has also been reported for the closely related *Streptomyces*-derived pentaene TPU-0043 (**4.22**) [244]. A 2019 study analysed the biosynthetic gene cluster [245], while a 2023 study reported that pentamycin yields could be increased through overexpression of two regulatory genes [246]. Pentamycin was first registered in Switzerland as Pentacin in 1980 for the treatment of vaginal fungal infections [121,122]. In August 2009, Lumavita AG obtained Swiss marketing authorisation for vaginal tablets of pentamycin (trade names Pruri-Ex and FemiFect) to treat vaginal candidiasis, trichomoniasis, and mixed infections [247,248]. The rights were transferred to Biolotus Biotechnology AG, but pentamycin was withdrawn from the market in 2015 [122].

Polyene MoA. Most polyene antifungal agents are amphipathic macrolides that act primarily by binding fungal ergosterol; their generally lower affinity for cholesterol contributes to selective antifungal activity, and these interactions underlie several overlapping MoA. In 1979, polyenes were divided into Groups I and II based on their relationships between K^+^ leakage, cell killing and haemolysis.

Group I. Polyenes such as natamycin (**4.12**) and pentamycin (**4.16**) induce K^+^ leakage and cell death or haemolysis at similar concentrations. For natamycin, however, subsequent work indicates that growth inhibition at pharmacologically relevant concentrations does not require appreciable membrane permeabilisation. Its antifungal activity is now thought to arise predominantly through binding to sterol-rich ordered membrane phases, perturbing ergosterol-dependent lipid packing and membrane-protein function rather than through classical ion-channel formation. Natamycin, the best-studied Group I member, interacts with sterol-induced liquid-ordered domains, disrupts phase separation and sterol dynamics, and inhibits ergosterol-dependent processes such as membrane and vacuole fusion without appreciable membrane permeabilisation at pharmacologically relevant concentrations [249,250,251].

Group II. Polyenes such as amphotericin B (**4.05**) and the nystatin complex components **4.01**–**4.04** induce substantial K^+^ leakage at low concentrations but require higher concentrations to induce cell death or haemolysis [252,253]. These antifungals assemble into extramembranous ‘sterol sponges’ that extract ergosterol from fungal membranes—a major contributor to their fungicidal activity—while a minor fraction can also form ergosterol-dependent ion channels that contribute additional membrane permeabilisation under some conditions [134,254,255]. Off-target cholesterol extraction is thought to contribute to amphotericin B’s dose-limiting nephrotoxicity in humans [256]. The related aromatic heptaene macrolides, such as candicidin and partricin, are proposed to share the same ‘sterol sponge’-dominated mechanism as amphotericin B: high-affinity complexes with ergosterol can aggregate and extract sterol from fungal membranes, with additional ion-channel formation [257].

Polyene resistance. Clinically significant resistance to polyene antifungals remains relatively uncommon overall, particularly in comparison with resistance to synthetic azoles. Nevertheless, reduced susceptibility or resistance is increasingly recognised in clinically important pathogens, including *C. auris* and other non-*albicans Candida* spp., *Aspergillus* spp. and some Mucorales [18,167,255]. Consistent with their sterol-targeting MoA, the principal resistance mechanisms involve changes in ergosterol biosynthesis, alterations in cell wall and membrane composition that reduce drug access to sterols, and enhanced stress-response pathways; the relative importance of these mechanisms varies among polyenes, fungal species and experimental or clinical settings. These changes often impose fitness costs, which may limit widespread dissemination of resistant strains.

For Group I polyenes, evidence is most extensive for natamycin (**4.12**). For this agent, clinical resistance remains rare and is mainly described in heavily exposed ocular or mucosal isolates, but available data point to altered sterol composition and membrane organisation as key drivers [258,259,260]. As natamycin acts by binding ergosterol-rich ordered membrane phases and perturbing lipid packing and membrane-protein function, resistance is thought to arise from reduced ergosterol or its replacement by alternative sterols that no longer support formation of the ordered domains required for natamycin binding, thereby blunting its lipid-phase-modulating effects at the cost of altered membrane physiology [261].

For Group II polyenes such as amphotericin B (**4.05**), resistance is most often linked to depletion or structural alteration of ergosterol via mutations or regulatory changes in ERG genes (e.g., *ERG2*, *ERG3*, *ERG5*, *ERG6* and *ERG11*), which diminish high-affinity binding and ‘sterol sponge’ activity but also compromise membrane function and virulence [7,18,255,261]. Additional contributions come from cell wall remodelling that restricts drug access and from upregulated oxidative-stress and stress-signalling pathways, including Hsp90/calcineurin networks [163]. In *C. auris*, within-host microevolution during therapy has been associated with a transition from amphotericin B-susceptible to -resistant phenotypes [163].

## 5. Griseofulvin

**Figure 5:**
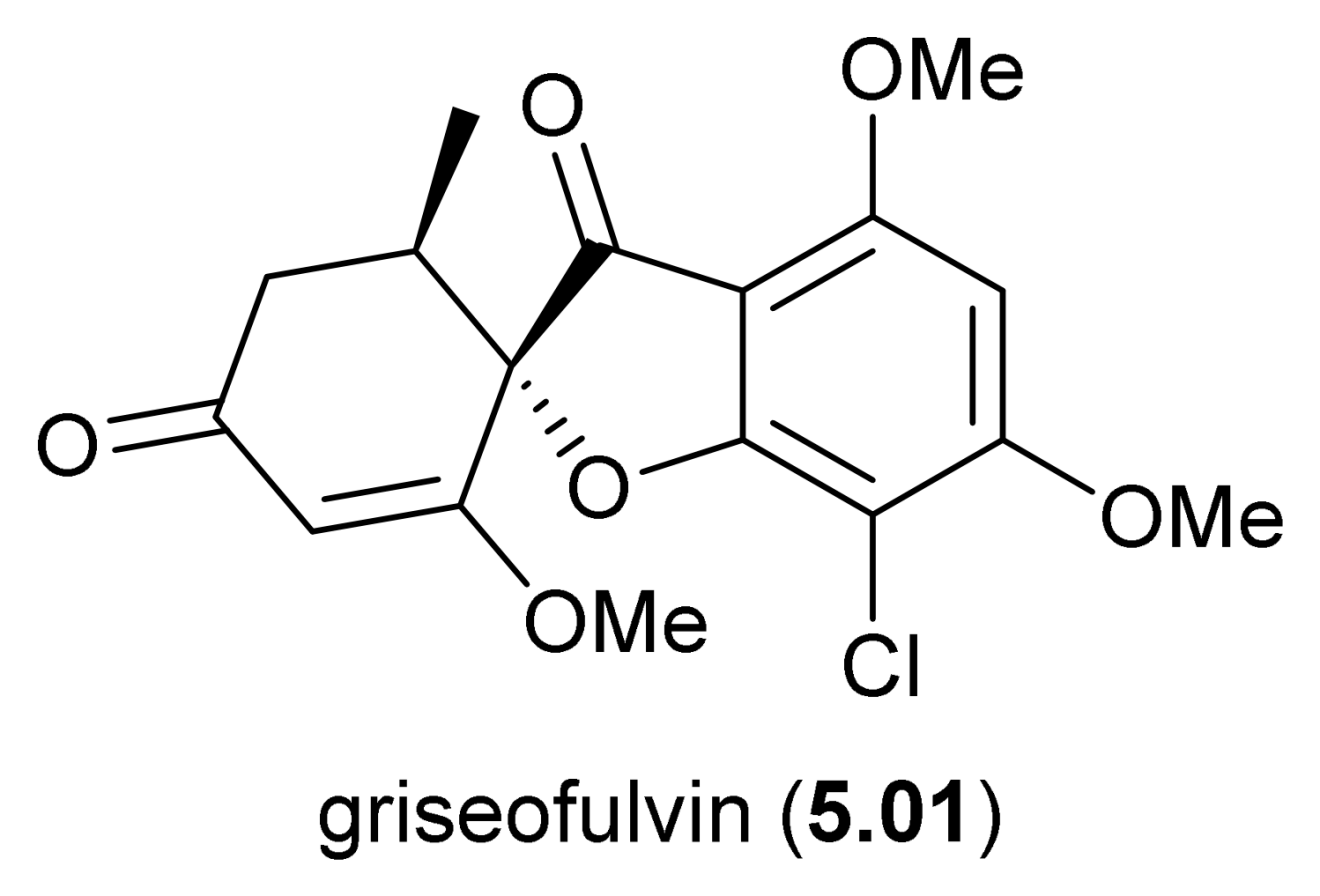
NP antifungal drug griseofulvin (**5.01**).

Griseofulvin (**5.01**) (Figure 5, Table 4) is a tricyclic polyketide first isolated from *Penicillium griseofulvum* in 1939 [262]. Its planar structure was correctly assigned in 1951 [263,264], while its relative and absolute stereochemistry were assigned using chemical and crystallographic methods in 1959 [265] and 1963 [266], respectively. Griseofulvin was independently recognised in the mid-1940s in *Penicillium janczewskii* as a fungal ‘curling factor’, owing to its capacity to induce abnormal curling of fungal hyphae [267,268]. Griseofulvin was launched in 1959 as an oral antifungal under the trade names Fulcin (ICI) and Grisovin (Glaxo) in the UK and Grifulvin (McNeil) and Grisactin (Ayerst) in the USA [36], only one year after demonstrating efficacy in the treatment of human ringworm infections [269,270]. Subsequent micronisation improved oral absorption, enabling equivalent blood concentrations at approximately half the prior dose [271]. Oral absorption is also enhanced by administration with a fatty meal [272,273]. Griseofulvin capsules and oral suspensions are still used to treat dermatophyte infections [274,275] and are included on the 2025 WHO Model List of Essential Medicines [49]. Its use has declined following the availability of faster-acting alternatives such as terbinafine and itraconazole, and access is now limited in some countries [276]. Griseofulvin was also historically used in agriculture as a crop protectant [277].

**Table 4:** Marketed griseofulvin antifungal drug.

Name	Origin	Year (Country) First Introduction/Approval [Ref.]	Pathogens/WHO	Admin.	Status
Griseofulvin (**5.01**)	NP	1959 (UK, USA) [36,278,279]	Der/WHO ^1^	O	C

^1^ Griseofulvin (**5.01**) was previously used as an agricultural crop protectant [277].

Griseofulvin MoA. Griseofulvin (**5.01**) is fungistatic and acts primarily by binding to tubulin and suppressing spindle-microtubule dynamic instability, including at microtubule plus ends. By reducing microtubule shortening and stabilising microtubules, it disrupts mitotic spindle function and arrests mitosis in susceptible dermatophytes, including *Trichophyton*, *Microsporum* and *Epidermophyton* spp. [280,281].

Griseofulvin resistance. Clinically significant resistance to griseofulvin (**5.01**) remains less well characterised than resistance to terbinafine and azoles, although reduced susceptibility and treatment failure have been reported, particularly following prolonged or suboptimal therapy [275,282]. Proposed and experimentally supported mechanisms include reduced intracellular drug accumulation, including through altered energy-dependent uptake and/or increased activity of ATP-binding cassette (ABC) transporters, as well as cell wall or morphological changes that may reduce effective intracellular drug exposure [283,284,285,286]. In the emerging dermatophyte *T. indotineae*, poor clinical responses to griseofulvin have been reported alongside frequent resistance to terbinafine and fluconazole; itraconazole and selected newer triazoles often retain in vitro activity [287,288].

## 6. Pyrithiones

**Figure 6:**
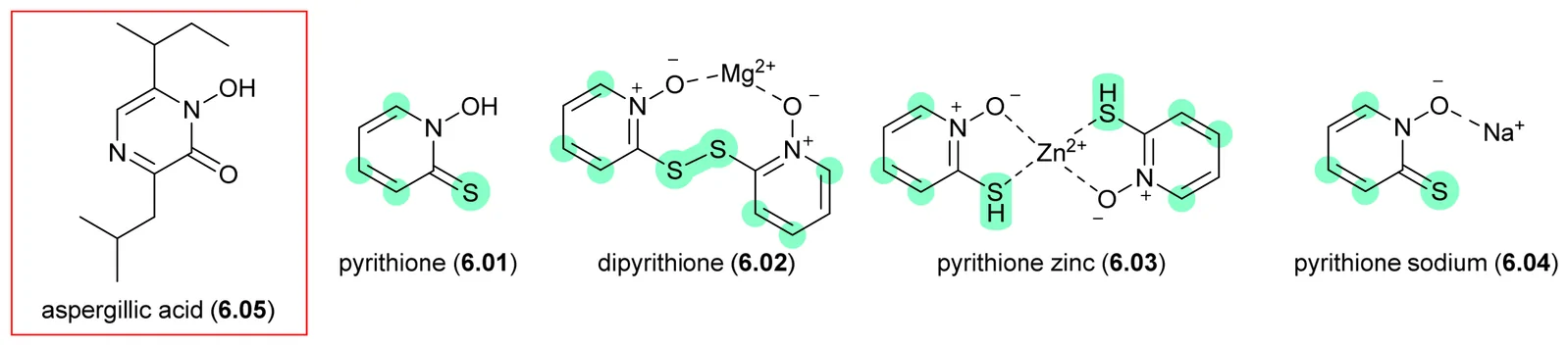
NP-D pyrithiones and salts **6.01**–**6.04** introduced for human use as antifungal agents. The NP aspergillic acid (**6.05**) is highlighted in red; structural modifications in pyrithiones **6.01**–**6.04** relative to aspergillic acid are highlighted in green.

Pyrithione (**6.01**) (Omadine) (Table 5, Figure 6) was synthesised in 1950 during investigations inspired by, or directed towards analogues of, the fungal natural product aspergillic acid (**6.05**) and shown to have potent antifungal and antibacterial activity [289,290]. Systemic development was not pursued because preclinical animal studies identified neurotoxicity and ocular toxicity at exposures that provided an inadequate safety margin for systemic administration [291]. However, this limitation could be circumvented by topical use. Procter & Gamble (USA) launched the anti-dandruff shampoo Head & Shoulders in 1961 [292], containing zinc pyrithione (**6.03**) as an active ingredient. Biosedra (France) also marketed sodium pyrithione (**6.04**), under the trade name Fonderma, as a topical treatment for dermatitis and seborrhoeic conditions [293]. Zinc pyrithione was prohibited for use in cosmetic products in the European Union (EU) from 1 March 2022 under Commission Regulation (EU) 2021/1902, following its classification as a category 1B reproductive toxicant.

**Table 5:** Pyrithiones introduced for human use as antifungal agents.

Name	Origin	Year (Country) First Introduction/Approval [Ref.]	Pathogens	Admin.	Status
Pyrithione (**6.01**) ^1^	NP-D	1961 (USA) [292]	Der, Mal	T	L
Dipyrithione (**6.02**)	NP-D	~1978 (USA) [294,295]	Der, Mal	T	D

^1^ Pyrithione was formulated as its zinc **6.03** and sodium **6.04** salts.

Dipyrithione (**6.02**) (omadine disulfide, bispyrithione magsulfex, Omadine MDS) was incorporated into several anti-dandruff shampoos as a magnesium complex manufactured by Olin Corporation (USA) from around 1978 [294,295]. Drossapharm (Switzerland) also marketed a magnesium dipyrithione complex in combination with undecylenic acid (**9.03**, Section 9), including an OTC tincture for seborrhoeic eczema of the scalp (Crimanex Tinktur; approved in 1987) and an anti-dandruff shampoo (Crimanex Shampoo; introduced in 1990) [296].

Pyrithione MoA. Pyrithione-containing metal complexes act primarily as a metal ionophore. In *Saccharomyces cerevisiae*, pyrithione-mediated copper influx triggers copper stress and inactivates essential iron–sulfur-cluster proteins [297,298]. In *Malassezia restricta*, zinc pyrithione increases intracellular zinc concentrations, with a smaller increase in copper, and is associated with mitochondrial dysfunction, impaired iron–sulfur-cluster synthesis and decreased lipase expression [299]. These effects, together with secondary perturbation of membrane potential and metal homeostasis, are consistent with its fungistatic activity.

Pyrithione Resistance. No reports describing clinically documented fungal resistance to pyrithione (**6.01**) were identified.

## 7. Echinocandins

**Figure 7:**
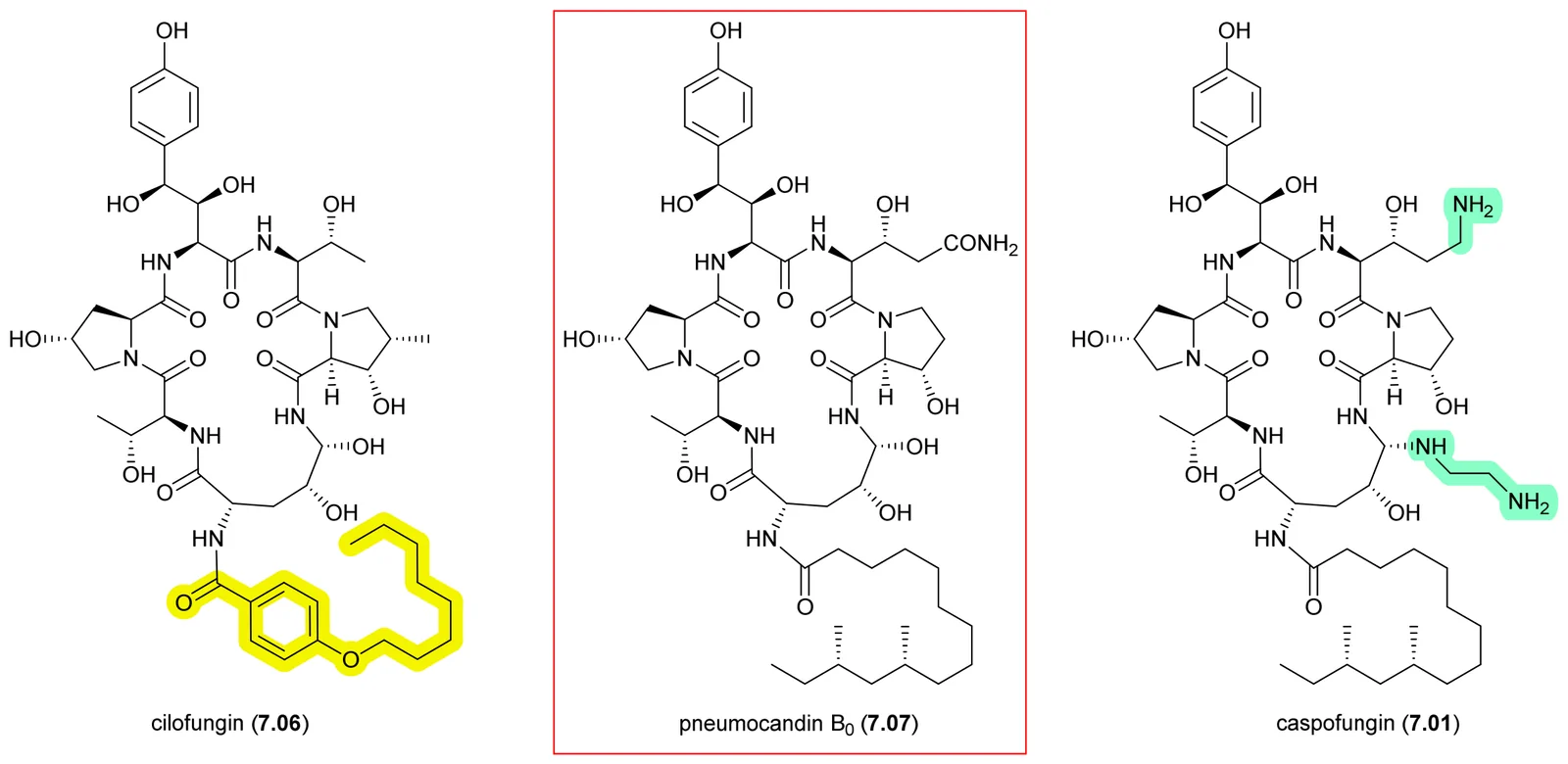
NP-D echinocandins: cilofungin (**7.06**), which entered clinical trials, and caspofungin (**7.01**), approved for human use as an antifungal drug. NP pneumocandin B0 (**7.07**) is highlighted in red, and structural modifications in caspofungin relative to pneumocandin B_0_ are highlighted in green. Structural modifications in cilofungin relative to NP echinocandin B (**7.05**) are highlighted in yellow; echinocandin B is shown in Figure 9.

**Figure 8:**
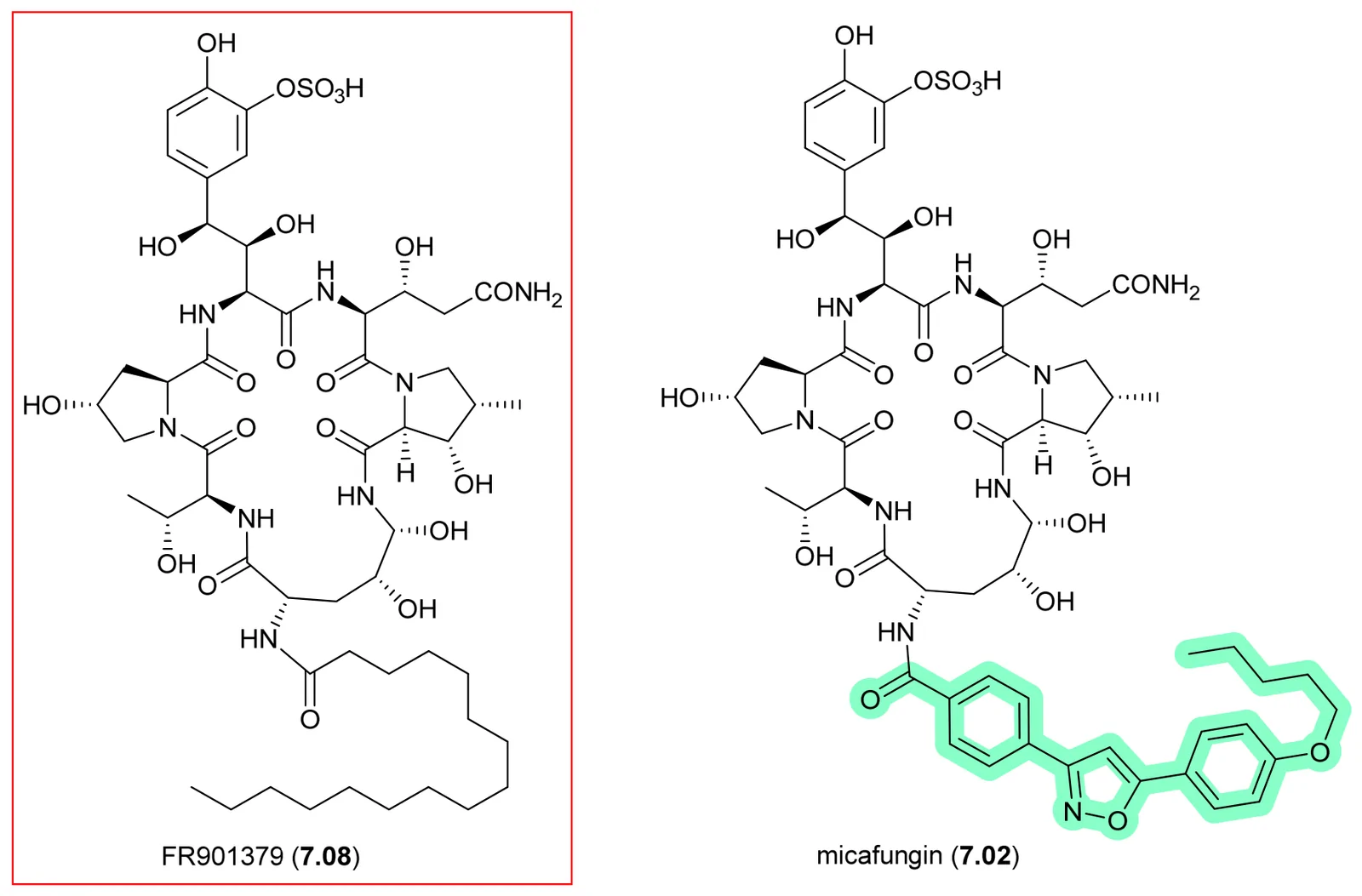
NP-D echinocandin antifungal micafungin (**7.02**), approved for human use. The NP FR901379 (**7.08**) is highlighted in red, and structural modifications in micafungin relative to FR901379 are highlighted in green.

**Figure 9:**
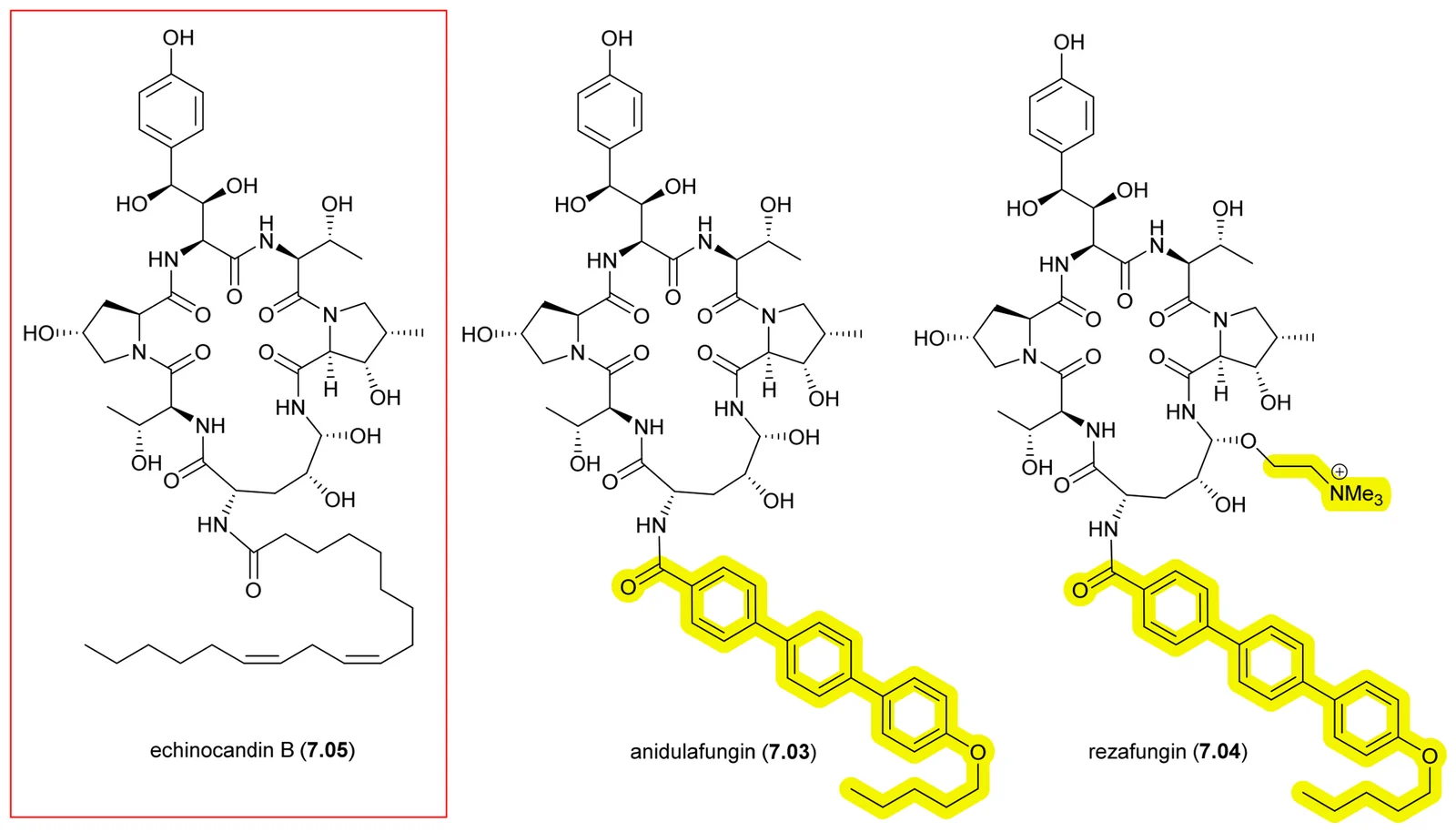
NP-D echinocandin antifungals anidulafungin (**7.03**) and rezafungin (**7.04**), approved for human use. The NP echinocandin B (**7.05**) is highlighted in red, and structural modifications relative to echinocandin B are highlighted in yellow.

In 1974, the first echinocandin, echinocandin B (**7.05**, Figure 9), was reported from several *Aspergillus* species, later reassigned to *Emericella* [300,301]. It displayed potent activity against yeasts, including *Candida* spp., but was ineffective against *C. neoformans* [302]. Since then, over 20 naturally occurring echinocandin analogues have been isolated [303,304]. The structure of echinocandin B was reported in 1976 as a cyclic hexapeptide core bearing a fatty acid side chain [303,305]. In 1984, the MoA of an analogue of echinocandin B was shown to be inhibition of β-1,3-D-glucan synthase [306]. Although this discovery spurred interest in developing this class, echinocandin B itself was unsuitable for clinical use owing to haemolysis at high doses, poor solubility, chemical instability and unfavourable protein binding [304]. Semisynthetic development was enabled by enzymatic deacylation of echinocandin B, which liberated an amine suitable for derivatisation and yielded non-haemolytic analogues, including cilofungin (**7.06**) (LY121019, Figure 7) [307]. Although cilofungin reached phase II clinical trials and demonstrated efficacy in oesophageal candidiasis, its development was discontinued in 1988 after the PEG-300 vehicle was associated with anion-gap metabolic acidosis and an increased risk of kidney injury [308]. Despite these setbacks, four semi-synthetic echinocandin drugs have since been launched: caspofungin (**7.01**), micafungin (**7.02**), anidulafungin (**7.03**) and rezafungin (**7.04**) (Figure 7, Figure 8 and Figure 9, Table 6).

**Table 6:** Approved echinocandin antifungal drugs.

Name	Origin	Year (Country) First Approval [Ref.]	Pathogens/WHO	Admin.	Status
Caspofungin (**7.01**)	NP-D	2001 (USA) [41]	Asp, Can/WHOc ^1^	IV	C
Micafungin (**7.02**)	NP-D	2002 (Japan) [42]	Asp, Can/WHOc ^1^	IV	C
Anidulafungin (**7.03**)	NP-D	2006 (USA) [43]	Asp, Can/WHOc ^1^	IV	C
Rezafungin (**7.04**)	NP-D	2023 (USA) [45]	Asp, Can	IV	C

^1^ Echinocandins are included on the complementary WHO Model List of Essential Medicines (WHOc), comprising medicines that require specialised diagnostic, treatment or monitoring facilities and/or specialist training, for safe and effective use [49].

Caspofungin (**7.01**) (Figure 7), developed by Merck & Co. (USA) under the trade name Cancidas, was the first echinocandin to obtain regulatory approval. In January 2001, it was approved in the USA for invasive aspergillosis in adults who were refractory to or intolerant of other antifungal therapies [41]. Caspofungin was subsequently approved by the FDA for oesophageal candidiasis (2002), invasive candidiasis (2003), and as an empirical antifungal therapy for febrile neutropenia (2004). Caspofungin is now approved and marketed globally, with generic formulations available. The history of the development of caspofungin has been reviewed in detail [309]. Pneumocandin B_0_ (**7.07**), one of several pneumocandins isolated from *Glarea lozoyensis* [310] (originally *Zalerion arboricola*) [311,312,313,314], became the starting point for extensive analogue synthesis and biological evaluation. These efforts ultimately yielded caspofungin, in which the 3-hydroxyglutamine side-chain amide was reduced to an amine, and the labile hemiaminal was converted to a stable ethylenediamine-containing substituent [309]. In the late 1980s, there was considerable interest in therapies for *Pneumocystis carinii* pneumonia—now termed *P. jirovecii* pneumonia (PJP)—a major opportunistic infection among people living with human immunodeficiency virus (HIV) [315]. Caspofungin has been evaluated as adjunctive or salvage therapy based on activity against pneumocystis cyst forms in experimental models [316], but is not considered a first-line agent for this indication [317,318].

Micafungin (**7.02**) (FK463, Figure 8), the second approved echinocandin, was developed by Fujisawa (now Astellas, Japan) and was approved in 2002 in Japan (as Fungard) for the treatment of *Candida* and *Aspergillus* infections [42]. Micafungin was approved under the trade name Mycamine in 2005 in the USA and in 2008 in Europe. The development of micafungin has been reviewed [319,320]. Briefly, FR901379 (**7.08**) (WF11899A), a sulfated echinocandin with enhanced aqueous solubility, was isolated from the fungus *Coleophoma empetri* [321,322] and served as the starting point for the discovery of micafungin [319,320]. Micafungin possesses a slightly bent side chain comprising a benzylcarboxylic-acid-substituted isoxazole linked to a *p*-pentyloxyphenyl group, a combination that confers potent in vivo activity against *Candida* and *Aspergillus* spp., high aqueous solubility and negligible haemolytic activity.

Anidulafungin (**7.03**) (LY303366, Figure 9) is a derivative of echinocandin B (**7.05**), developed by Lilly Research Laboratories (USA), with a linear side chain comprising a benzylcarboxylic-acid moiety linked via a benzyl group to a *p*-pentyloxyphenyl substituent [304,323,324,325]. An oral formulation entered clinical development soon after cilofungin’s clinical failure; however, Eli Lilly discontinued development of anidulafungin after a phase II trial demonstrated poor oral bioavailability [326]. Anidulafungin was licensed to Versicor in 1999; Versicor later merged to form Vicuron Pharmaceuticals, which was subsequently acquired by Pfizer (USA) in 2005. In February 2006, Pfizer obtained FDA approval for an IV formulation of anidulafungin (Eraxis) to treat candidaemia and other invasive *Candida* infections, followed by a paediatric indication expansion in September 2020. In September 2007, the European Medicines Agency (EMA) authorised anidulafungin for the treatment of candidiasis in adults and children greater than 1 month old under the trade name Ecalta. Anidulafungin is available in many countries globally, both as originator brands and increasingly as generics or alternative branded generics in markets such as Australia, Europe and India.

Rezafungin (**7.04**) (CD101, Figure 9) is an anidulafungin-derived echinocandin developed by Cidara Therapeutics (USA) to provide enhanced chemical stability, an ultra-long half-life and once-weekly IV dosing, while retaining potent antifungal activity against *Candida* and *Aspergillus* spp. [304,327,328]. Specifically, the hemiaminal was derivatised with a quaternary trimethylethylamine group, increasing the adult human half-life of rezafungin to approximately 130 h, compared with approximately 40–50 h for anidulafungin and caspofungin and 11–21 h for micafungin [329]. Cidara entered a licence agreement with Melinta Therapeutics (USA) for exclusive US commercial rights for rezafungin after Cidara submitted a New Drug Application (NDA) to the FDA. Rezafungin was approved by the US FDA in March 2023 under the trade name Rezzayo for adults with limited or no alternative treatment options for candidaemia and invasive candidiasis [330]. Melinta launched and marketed rezafungin in the USA until CorMedix Therapeutics (USA) acquired Melinta in September 2025, after which CorMedix assumed US marketing. Mundipharma (UK) originally licensed commercial rights outside the USA and Japan from Cidara in September 2019 and acquired the remaining global assets and rights from Cidara in April 2024. This asset acquisition followed Rezzayo approvals in the EU (December 2023), the UK (January 2024) and the UAE (March 2024) [331].

Echinocandin MoA. Echinocandins inhibit the catalytic Fks subunits of the fungal β-1,3-D-glucan synthase enzyme complex, principally Fks1p, and in some species or physiological states, Fks2p [332,333]. This glucosyltransferase polymerises UDP-glucose into β-1,3-glucan chains, the major structural polysaccharide of the fungal cell wall. Inhibition disrupts cell wall integrity, leading to osmotic instability and fungal cell lysis, which typically results in fungicidal activity against *Candida* spp. and fungistatic activity against *Aspergillus* spp. [334]. The fungerp antifungal ibrexafungerp (**8.01**) (Section 8) targets the same enzyme through a non-identical, partially overlapping Fks binding site, consistent with its retained activity against many, but not all, echinocandin-resistant strains [333].

Echinocandin resistance. Clinical resistance has been reported in *C. albicans*, *Nakaseomyces glabrata* (formerly *C. glabrata*), *Pichia kudriavzevii* (formerly *C. krusei*), *C. tropicalis* and *Clavispora lusitaniae* (formerly *Candida lusitaniae*) [334]. By contrast, resistance remains uncommon in *A. fumigatus*, possibly because resistance-associated *FKS1* mutations impose a substantial in vivo fitness cost [335,336]. Resistance is usually associated with mutations in *FKS1* in *Candida* spp.; in *N. glabrata,* mutations in *FKS2* may also arise. The most clinically important mutations cluster in three conserved ‘hot spot’ regions in Fks1/Fks2, which are brought into proximity in the folded protein near the transmembrane drug-binding region. In an experimental *S. cerevisiae* model, S643P in Fks1 hotspot 1 markedly reduces echinocandin susceptibility. Cryogenic electron microscopy (cryo-EM) structures of wild-type *S. cerevisiae* Fks1 and the echinocandin-resistant Fks1(S643P) mutant indicate that altered local lipid arrangements around the affected transmembrane region may diminish echinocandin binding and/or alter the enzyme’s drug-induced conformational response while preserving β-1,3-glucan synthase activity [333,337,338]. Compensatory cellular responses, including upregulation of chitin synthesis and stress-response pathways, can partially stabilise the cell wall under drug pressure and facilitate survival or tolerance while FKS mutations emerge [332]. Intrinsic reduced susceptibility in some fungi is linked to naturally occurring *FKS* polymorphisms that diminish drug binding [332]. Echinocandins appear to be poor substrates for the major fungal drug-efflux systems; consequently, adaptive efflux is not regarded a major established resistance mechanism.

## 8. Fungerp

**Figure 10:**
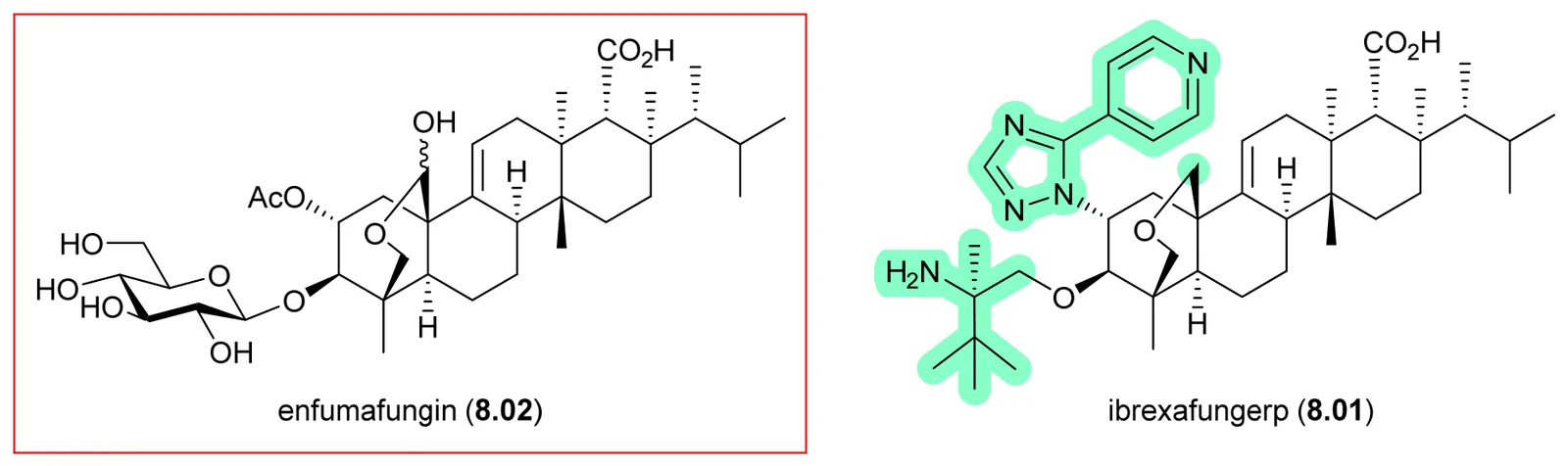
NP-D fungerp antifungal ibrexafungerp (**8.01**), approved for human use. The NP progenitor enfumafungin (**8.02**) is outlined in a red box, and structural modifications in ibrexafungerp relative to enfumafungin are highlighted in green.

Enfumafungin (**8.02**) (Figure 10) is a fernane-type triterpene glycoside [339,340] isolated from the endophytic fungus *Hormonema carpetanum* and displays potent activity against *Candida* and *Aspergillus* spp. [341,342,343,344,345]. Despite this promising antifungal profile, enfumafungin had pharmacokinetic properties unsuitable for further clinical development [346]. Extensive structure-activity relationship (SAR) studies at Merck & Co. (USA), and later SCYNEXIS (USA), investigated modification of (i) the hemiacetal to an ether, (ii) replacement of the d-glucose with ethanolamine-containing derivatives, and (iii) the C-2 acetate to nitrogen-containing heterocycles [346,347,348,349,350]. From these efforts, ibrexafungerp (**8.01**) (Table 7) was selected for development, and after successful clinical trials it gained US FDA approval for two indications (trade name Brexafemme): oral tablets for vulvovaginal candidiasis (VVC; June 2021) and for reduction in the incidence of recurrent VVC (RVVC) in November 2022 [44]. Ibrexafungerp is distinguished from echinocandins by oral bioavailability and by retained activity against many, though not all, echinocandin-resistant isolates [351,352]. A potential manufacturing cross-contamination risk was identified because equipment used for ibrexafungerp citrate had also been used to manufacture a non-antibacterial β-lactam-containing drug substance. In response, SCYNEXIS initiated a voluntary recall of Brexafemme and temporarily suspended ibrexafungerp clinical studies while a mitigation strategy and resupply plan were developed [353]. No contamination of marketed ibrexafungerp product or associated adverse events had been confirmed in the reported account. Ibrexafungerp was subsequently licensed to GlaxoSmithKline (GSK, UK) under an exclusive agreement covering commercialisation of Brexafemme and further clinical development [354]. Although GSK has indicated an intention to restore availability, no firm public relaunch date had been identified as of August 2026 [355]. No regulatory approvals outside the USA were identified.

**Table 7:** Marketed fungerp antifungal drug.

Name	Origin	Year (Country) First Approval [Ref.]	Pathogens	Admin.	Status
Ibrexafungerp (**8.01**)	NP-D	2021 (USA) [44]	Can	O	L ^1^

^1^ Ibrexafungerp (**8.01**) retains US regulatory approval but was voluntarily recalled from the US market because of a potential manufacturing cross-contamination issue. Commercial supply was interrupted, and no confirmed relaunch date had been announced as of August 2026.

Fungerp MoA. Ibrexafungerp (**8.01**) and enfumafungin (**8.02**), as well as the echinocandins (Section 7), inhibit β-1,3-d-glucan synthase [341,342]. The membrane-integrated Fks complex catalyses the synthesis and translocation of β-1,3-D-glucan across the plasma membrane for incorporation into the fungal cell wall [333]. Cryo-EM structures of *S. cerevisiae* Fks1 and the echinocandin-resistant Fks1(S643P) mutant support the existence of non-identical, partially overlapping Fks interaction sites for fungerps and echinocandins [337,338]. This difference is consistent with limited target-based cross-resistance [356].

Fungerp resistance. Clinical resistance to ibrexafungerp (**8.01**) has been reported infrequently to date, reflecting in part its relatively recent introduction and use. Clinical and laboratory studies have shown that reduced ibrexafungerp susceptibility can arise in *Candida* spp. via mutations in *FKS1*, as well as *FKS2* in *N. glabrata* [351,352,357,358]. The extent of cross-resistance with echinocandins is substitution-dependent, because ibrexafungerp and echinocandins engage non-identical, partially overlapping regions of glucan synthase. Consequently, many isolates with echinocandin-resistance-associated *FKS* mutations retain low ibrexafungerp susceptibility, whereas particular *FKS* substitutions can reduce susceptibility to one or both drug classes.

## 9. Fatty Acids

Fatty acids are ubiquitous naturally occurring compounds, some of which possess antifungal and antiseptic activity (Table 8). Since the late nineteenth century, several stearic acid (**9.01**) salts have been used as antiseptics or disinfectants, including sodium stearate (1889), zinc stearate (1894) and magnesium stearate (1900) [46]. The 2019 Lost Medicines analysis described zinc stearate as an antifungal and anti-acne agent [46]; however, zinc ions may have contributed to these reported effects [359].

**Table 8:** Fatty-acid antiseptic and antifungal drugs introduced for human use.

Name	Origin	Year (Country) First Introduction/Approval [Ref.]	Pathogens	Admin.	Status
Stearic acid (**9.01**) ^1^	NP	1889 (Germany, USA) [46]	Antiseptic, Der	T	D
Caprylic acid (**9.02**) ^2^	NP	1945 (USA) [36]	Der	T	D
Undecylenic acid (**9.03**) ^3^	NP-D	1945 (USA) [36]	Der	T	C
Pelargonic acid (**9.04**)	NP	1960 (USA) [36]	Der ^4^	T	D

^1^ Stearic acid (**9.01**) was used as various stearate salts: sodium (first used 1889), zinc (1894) and magnesium (1900). ^2^ Marketed as sodium caprylate. ^3^ Undecylenic acid has also been used in combination with dipyrithione (**6.02**) in a tincture for scalp seborrhoeic eczema and in anti-dandruff shampoos (Section 6). ^4^ Pelargonic acid (**9.04**) remains in use principally in crop-protection and food-related applications, including as a food additive and in commercial fruit- and vegetable-peeling solutions.

**Figure 11:**
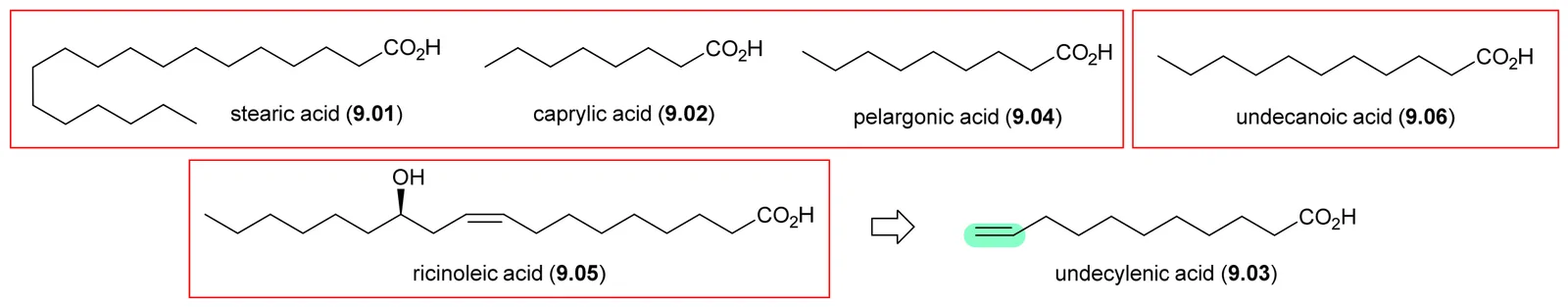
Antiseptic stearic acid (**9.01**) and the medium-chain fatty acid antifungal drugs **9.02**–**9.04**, along with undecanoic acid (**9.06**) used in several MoA and resistance studies. The naturally occurring fatty acids **9.01**, **9.02**, **9.04**–**9.06** are highlighted in red. Structural modification of castor oil-derived ricinoleic acid (**9.05**) to yield the NP-D undecylenic acid (**9.03**), used industrially for its manufacture, is highlighted in green.

In 1938, Peck and Rosenfeld reported that human sweat possesses fungicidal properties attributable to its fatty acid content and noted that these effects occurred without associated irritation [360] This prompted a systematic study that found the sodium salts of undecylenic acid (**9.03**), caprylic acid (**9.02**) and pelargonic acid (**9.04**) (Figure 11) were particularly active against the dermatophyte *Trichophyton gypseum* (now classified as *Nannizzia gypsea* [361]), at neutral to mildly alkaline pH, while fatty acids with six or fewer carbon atoms showed greater potency at more acidic pHs [360]. These observations were consistent with a 1913 report of the activity of fatty acids against *Aspergillus niger* [362] and were followed by several studies examining dermatophytes and other pathogenic fungi [363,364,365,366].

Pennwalt Corporation (USA) introduced caprylic acid (**9.02**) (octanoic acid) into the US market in 1945 under the trade name Sodium Caprylate as a topical treatment for dermatomycoses [36,366]. Caprylic acid occurs in human, bovine and goat milk and in cuphea, coconut and palm-kernel oils [367]. Clinical use of sodium caprylate appears to have been relatively brief.

In 1945, Pennwalt introduced undecylenic acid (**9.03**; 10-undecenoic acid) in the USA as Desenex, a topical treatment for athlete’s foot and other dermatomycoses [36]. Early clinical evaluations of undecylenic acid preparations were undertaken largely by the US military during the Second World War [368,369,370,371,372]. Initial topical formulations contained 5% undecylenic acid and 20% zinc undecylenate [369,371], whereas later Desenex formulations used other component ratios or, in some products, substituted undecylenic acid with miconazole nitrate (2%). In 1965, Pennwalt introduced the calcium salt under the trade name Cruex [36] as a topical spray powder for tinea cruris formulated with calcium undecylenate (10–15% *w*/*w*) in a talc-based, perfumed powder vehicle. However, calcium undecylenate had been used in other formulations from 1961 [373]. Undecylenic acid-based formulations remain widely available as OTC treatments for athlete’s foot and other superficial dermatomycoses. Undecylenic acid has also been used in combination with dipyrithione (**6.02,** Section 6) in a tincture for seborrhoeic eczema of the scalp and in an anti-dandruff shampoo [296]. Although trace-level occurrence of undecylenic acid has been reported in two plant studies using GC–MS [374,375], it is classified here as NP-D because marketed undecylenic acid is manufactured industrially from ricinoleic acid (**9.05**), the most abundant ω-9 hydroxy fatty acid in castor oil [376,377]. Thermal cleavage affords undecylenic acid and heptanal, followed by isolation and purification of the desired acid. Undecylenic acid has often been described in secondary sources as a natural constituent of human sweat; however, we did not identify a primary study directly demonstrating its presence in sweat. The apparent confusion may derive from early reports attributing the fungistatic activity of sweat to its free fatty acid fraction, followed by testing of commercially available fatty acids, including undecylenic acid [360,378].

In 1960, pelargonic acid (**9.04**) (nonanoic acid) was introduced in the USA as the active component of the topical antifungal preparation Pellar, developed by Crookes-Barnes (USA) [36]. Pelargonic acid occurs at low levels in nature, mainly as a minor constituent of various plant essential oils and animal lipids [379]. Pellar appears to have been marketed in two formulations: a cream with 4% pelargonic acid, 10% zinc pelargonate and 2% finely divided sulfur, and a talc-based powder with 2.5% pelargonic acid, 20% zinc pelargonate and 3% zinc sulfide [380]. Although pelargonic acid appears to have had limited uptake as a human topical antifungal, it remains in use as a bioherbicide [379], as well as a flavouring agent and food additive, and in cosmetic, personal care and cleaning products [381].

Fatty acid MoA. Caprylic acid (**9.02**) and pelargonic acid (**9.04**) have been shown to perturb fungal membranes [382,383,384,385]. Studies of these and related medium-chain fatty acids have also reported coordinated transcriptional remodelling of membrane- and cell-wall-associated pathways, consistent with impaired dermatophyte fitness and viability [386]. Undecylenic acid (**9.03**) inhibits *C. albicans* germ-tube formation at concentrations that do not substantially inhibit growth, consistent with a morphogenesis-selective effect; disruption of lipid metabolism and/or cytoplasmic alkalinisation was proposed but not established [387]. In a separate *C. albicans* biofilm study [388], undecylenic acid reduced biomass, filamentation and metabolic activity, caused abnormal cell-surface ultrastructure, and altered expression of genes associated with virulence, adhesion and hyphal development. The most detailed mechanistic studies have been undertaken on the saturated *n*-C11 fatty acid, undecanoic acid (**9.06**). In *Trichophyton rubrum*, undecanoic acid weakly inhibits growth and suppresses secretion of virulence-associated lipases and keratinases required for host-tissue invasion [365,389]. It is incorporated into fungal lipid metabolism and is associated with oxidative stress, disruption of fatty acid, phospholipid and ergosterol synthesis, and coordinated transcriptional responses that remodel the membrane and cell wall, collectively compromising dermatophyte viability [389,390,391]. These findings provide a mechanistic framework for related medium-chain fatty acids, but whether the same processes contribute to the activity of the marketed agents remains to be established.

Fatty acid antifungal resistance. Although no well-characterised clinical resistance mechanisms have been identified for the marketed fatty acid antifungals, fungal responses to the closely related medium-chain fatty acid undecanoic acid (**9.06**) appear to involve metabolic adaptation, efflux and stress defence rather than a single defined target-site mutation [365]. In *T. rubrum*, undecanoic acid can be incorporated into phospholipids and subsequently metabolised through induced β-oxidation pathways, while antioxidant defences are upregulated in response to oxidative stress [391]. Transcriptomic studies have also identified condition-dependent changes in expression and splicing of genes encoding ATP-binding cassette (ABC) and major facilitator superfamily (MFS) transporters [392,393,394]. These findings are consistent with an adaptive efflux-associated tolerance response, although their causal contribution to clinically relevant resistance remains unproven.

In experimental systems, resistance or tolerance phenotypes have also been associated with mutations in genes such as *udaA* and *lipA* in *A. nidulans* [395,396], chromosome-7 copy-number variation in a laboratory-derived *C. albicans* strain (rather than *DAL81* deletion itself) [397] and biofilm-associated tolerance in *Candida* spp. [398]. Well-characterised resistance mechanisms have not been reported for caprylic acid (**9.02**) and pelargonic acid (**9.04**). Serial in vitro passage with undecylenic acid (**9.03**) was reported in 1955 to select a low-level resistant *T. mentagrophytes* phenotype [399], but its molecular basis and clinical relevance remain unknown.

## 10. Antimetabolite

**Figure 12:**
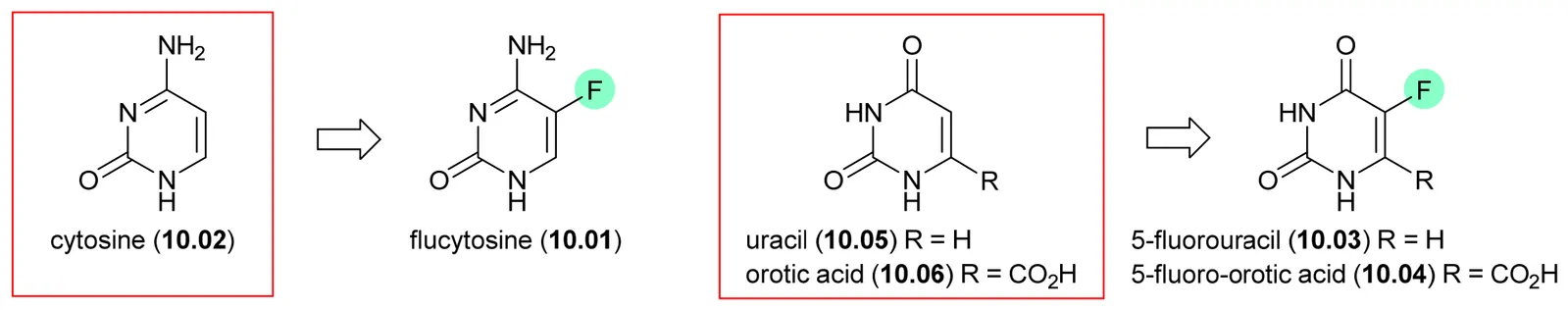
The NP-D antifungal antimetabolite flucytosine (**10.01**), approved for human use, and the anticancer NP-D antimetabolites 5-fluorouracil (**10.03**) and 5-fluoro-orotic acid (**10.04**). The naturally occurring cytosine (**10.02**), uracil (**10.05**) and orotic acid (**10.06**) are highlighted in red. Structural modifications in flucytosine relative to cytosine, 5-fluorouracil relative to uracil and 5-fluoro-orotic acid relative to orotic acid are highlighted in green.

Flucytosine (**10.01**; 5-fluorocytosine, 5-FC, Ro 2-9915) (Table 9, Figure 12) is a fluorinated cytosine analogue derived from cytosine (**10.02**). It was first reported in 1957 by Hoffmann-La Roche (Switzerland) during a programme seeking new anticancer agents [400]. The same study described the synthesis of 5-fluorouracil (**10.03**) and 5-fluoro-orotic acid (**10.04**), analogues of uracil (**10.05**) and orotic acid (**10.06**), respectively [400]. Although 5-fluoro-orotic acid showed in vivo antitumour activity and 5-fluorouracil was subsequently developed as an anticancer drug, flucytosine was inactive in the original tumour models [401,402]. Flucytosine was subsequently recognised as a potent antifungal, with activity in experimental models of candidiasis, cryptococcosis and chromoblastomycosis [403,404,405], a chronic cutaneous and subcutaneous fungal infection [406]. Flucytosine was first approved in the USA in November 1971 under the trade name Ancobon for treatment of severe *Candida* and *Cryptococcus* infections [36,407,408]. A comprehensive review of flucytosine’s discovery, pharmacology, clinical indications, pharmacokinetics, toxicity and drug interactions was published by Vermes and co-workers in 2000 [405], with an updated clinical review more recently provided by Sigera and Denning [409]. Historically, flucytosine was supplied as oral tablets or capsules and, in some markets, as an IV formulation (Ancotil); however, availability of the IV formulation is regionally limited rather than its use being uniformly infrequent. Flucytosine is now used predominantly, but not exclusively, in combination regimens, most importantly with amphotericin B (**4.05**) (Section 4), including liposomal amphotericin B (**4.05**, Section 4), or high-dose fluconazole [405,409,410,411]. These combinations are important components of induction therapy for cryptococcal meningitis and for selected severe *Candida* infections; the agents are supplied as separate products rather than fixed-dose combinations. Oral and IV formulations of flucytosine are listed on the 2025 WHO Model List of Essential Medicines for the treatment of cryptococcosis [49]. High flucytosine exposure, particularly in the setting of impaired renal clearance, is associated with dose-related bone-marrow suppression, hepatotoxicity and gastrointestinal adverse effects; nephrotoxicity may be exacerbated by co-administration with amphotericin B [409,411]. Lower-dose flucytosine regimens have relatively low rates of severe toxicity, although idiosyncratic reactions such as rash, mucosal lesions, and nausea still occur.

**Table 9:** Antimetabolite antifungal drug approved for human use.

Name	Origin	Year (Country) First Approval [Ref.]	Pathogens/WHO	Admin.	Status
Flucytosine (**10.01**)	NP-D	1971 (USA) [36,407,408]	Can, Cry/WHO ^1^	O, IV	C

^1^ Flucytosine (**10.01**) has also been used in veterinary medicine, predominantly on an off-label basis in most jurisdictions.

Flucytosine MoA. Flucytosine (**10.01**) is a prodrug that first enters fungal cells through a cytosine permease, principally Fcy2, and is deaminated by fungal cytosine deaminase, Fcy1, to 5-fluorouracil (**10.03**) [411,412]. 5-Fluorouracil is converted by uracil phosphoribosyltransferase, Fur1, to 5-fluorouridine monophosphate, which is further metabolised to 5-fluorouridine triphosphate and incorporated into RNA in place of uridine nucleotides, impairing protein synthesis. Metabolic conversion also generates 5-fluorodeoxyuridine monophosphate, which inhibits thymidylate synthase and thereby compromises DNA synthesis. Humans lack cytosine deaminase; nevertheless, conversion of flucytosine to 5-fluorouracil by intestinal microorganisms has been proposed as one contributor to host toxicity [405,409]. In clinical practice, serious adverse effects are most closely associated with elevated systemic flucytosine concentrations, particularly in renal impairment, underscoring the importance of dose adjustment and therapeutic drug monitoring.

**Flucytosine resistance.** Use of flucytosine (**10.01**) is constrained by frequent primary and acquired resistance, particularly during monotherapy [413]. Resistance most commonly results from mutation or loss of pyrimidine salvage pathway components required for flucytosine uptake and activation: cytosine permease (Fcy2; reduced uptake), cytosine deaminase (Fcy1; impaired conversion to 5-fluorouracil) and uracil phosphoribosyltransferase (Fur1; impaired generation of fluorinated nucleotide metabolites) [411,414]. Increased *de novo* pyrimidine biosynthesis can also reduce dependence on the salvage pathway and thereby diminish flucytosine susceptibility. Additional mechanisms reported in experimental or genomic studies of *Candida* and *Cryptococcus* spp. include altered expression or function of permease and deaminase paralogues, together with adaptive cell-wall remodelling that may contribute to tolerance or reduced susceptibility [411,413,415,416].

## 11. Data Analysis

**Figure 13:**
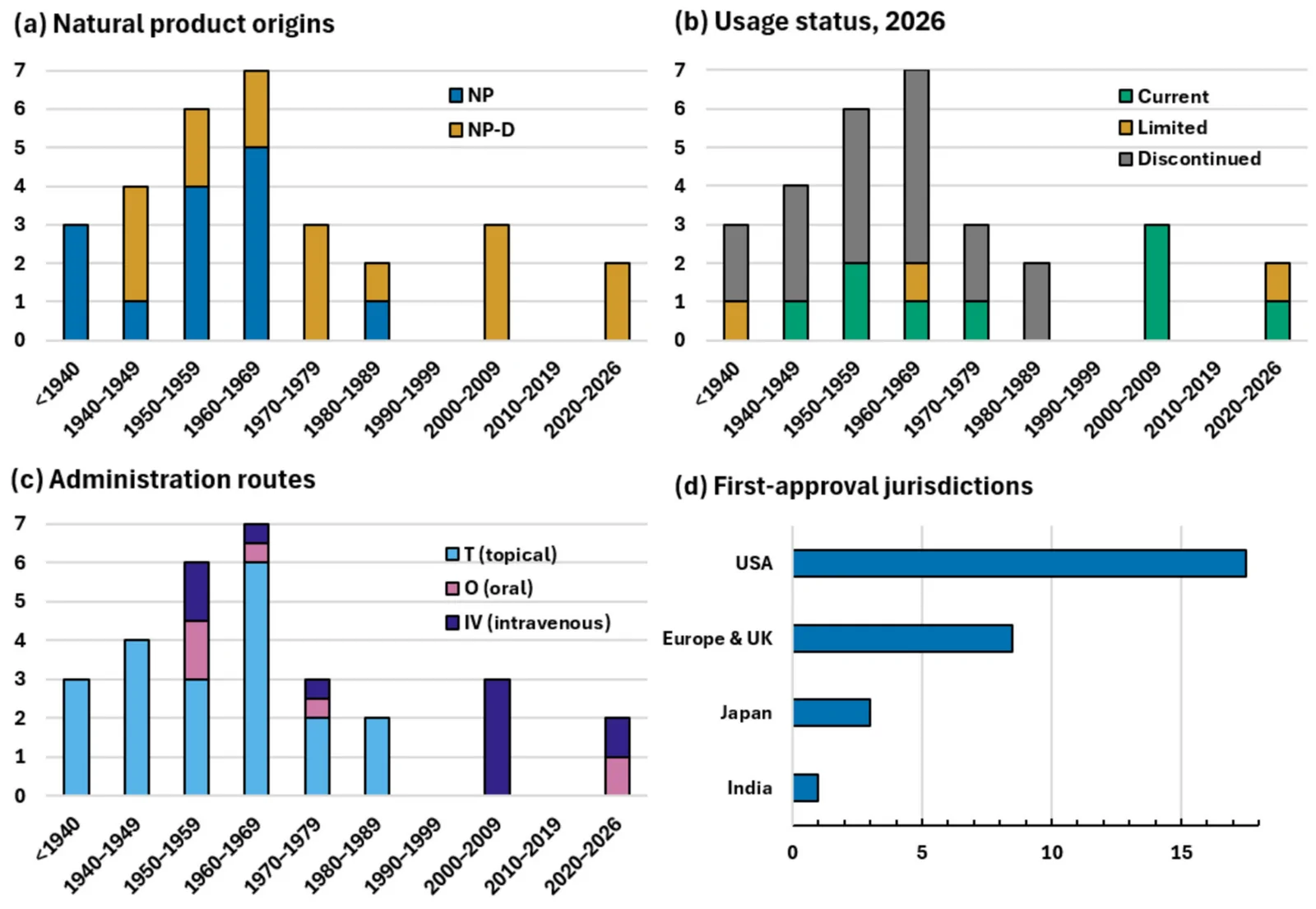
Historical distribution of 30 natural product (NP) and natural product-derived (NP-D) antifungal drugs or drug complexes introduced or approved for human use. For several early agents, the date and jurisdiction indicate the earliest substantiated documentary record of introduction rather than a formal marketing authorisation, and some years are approximate; the figure therefore depicts broad historical patterns rather than precise temporal counts or modern regulatory status. (**a**) First introductions or approvals by decade, classified by origin. (**b**) Usage status as of August 2026: current human antifungal use, limited availability or discontinued. (**c**) Route of administration. Each therapeutic entity contributes a total weight of 1.0. Where an entity was introduced for use by two recorded routes, a weight of 0.5 was allocated to each route, thereby avoiding double counting and preserving the total of 30 therapeutic entities in each decade. (**d**) Jurisdiction of first documented introduction or approval. Each therapeutic entity contributes a total weight of 1.0. Where the earliest documented introduction occurred in two jurisdictions in the same year, a weight of 0.5 was allocated to each jurisdiction. Europe/UK combines continental European countries and the UK, including historical introductions or uses that predate modern regulatory-approval systems.

Since the introduction of benzoic acid (**3.01**) in 1872, a total of 30 NP-inspired antifungal drugs or drug complexes have been introduced or approved for human use, comprising 14 NPs and 16 NP-D drugs (Figure 13a, Supplementary Materials File S1).

The first documented introductions or approvals of new NP-inspired antifungals peaked in the 1960s and, by 1980, had largely stalled. Of the 25 NP-inspired antifungals introduced or approved prior to 1981, five remain in current use as of August 2026; two have limited availability, and 18 are discontinued (Figure 13b). Ibrexafungerp (**8.01**) was classified as having limited availability because, although US regulatory approval remained in force, commercial supply was voluntarily interrupted, and no firm public relaunch date had been identified at the August 2026 cut-off.

Of particular concern, only five NP-inspired antifungals have been approved in the 45 years since 1981: the echinocandins caspofungin (**7.01**), micafungin (**7.02**), anidulafungin (**7.03**) and rezafungin (**7.04**) and the fungerp ibrexafungerp (**8.01**). All inhibit fungal β-1,3-D-glucan synthase, although ibrexafungerp binds a non-identical, partially overlapping Fks site relative to the echinocandins. This narrow concentration of recent approvals around the β-1,3-glucan-synthase complex underscores the need for agents with differentiated molecular targets and mechanisms. Reformulation of existing NP-inspired antifungals also remains valuable, as demonstrated by liposomal amphotericin B (**4.05**, Section 4), which substantially reduced the infusion-related reactions and renal toxicity associated with amphotericin B deoxycholate and is widely used for treatment of several invasive mycoses.

Figure 13c shows a transition from predominantly topical or local administration in the earliest approval periods towards a greater contribution from IV and oral administration among later agents. This pattern is consistent with an early emphasis on superficial dermatological and mucosal infections, whereas later approvals increasingly addressed invasive fungal disease and therefore required systemic administration.

Finally, Figure 13d shows that first documented introductions or approvals were concentrated in the USA and Europe/UK, which together account for 26 of the 30 agents represented. This distribution likely reflects the historical concentration of pharmaceutical development, surviving documentation, and regulatory records in these jurisdictions; it should not be interpreted as a measure of current availability or of the geographic origin of the underlying NPs.

## 12. Conclusions

NP-inspired antifungals (NP and NP-D) have clearly delivered enormous benefits to modern healthcare. That said, the number and chemical diversity of approvals, and the number that remain in current use, fall short of those achieved over the same time period for NP-inspired antibacterial drugs [28], although the two fields share several historical and developmental trends. Notwithstanding uncertainty in some early introduction dates and jurisdictions, the compiled historical record suggests that NP-inspired antifungal introductions and approvals were concentrated in the mid-twentieth century, particularly during the 1960s, and became much less frequent from the 1970s onwards.

Moreover, with only nine of the 30 NP-inspired antifungal drugs or drug complexes remaining in current use as of August 2026, and three additional agents having limited availability, the increasing prevalence of drug-resistant fungal pathogens reinforces the clear and urgent need for investment in antifungal discovery and development. A WHO analysis identified 43 systemic antifungal products in clinical or preclinical development [19] as of September 2024, but concluded that the pipeline remains insufficient to meet the needs posed by priority fungal pathogens [417]. Recent NP and NP-D antifungal candidates in clinical development have been reviewed elsewhere [418].

This gap is particularly important because the five NP-inspired agents introduced or approved since 1981, the echinocandins and ibrexafungerp, all target β-1,3-D-glucan synthase. Encouragingly, the last 25 years have coincided with remarkable advances in multidisciplinary microbial biodiscovery sciences, ranging from genome sequencing and annotation, which can reveal otherwise cryptic biosynthetic gene clusters, to advances in analytical, natural product, organic and medicinal chemistry, as well as the development of more informative bioassays and greater recognition of the need to identify and validate new fungal molecular targets.

The past eight decades demonstrate that microbial natural products can provide chemical starting points for valuable antifungal medicines. The challenge now is to translate the expanding capabilities of contemporary biodiscovery into structurally diverse, clinically useful agents with differentiated mechanisms of action. Past achievements should therefore serve not only as a historical record, but also as a rationale for renewed and sustained investment in antifungal NP discovery and development.

## Figures and Tables

**Table 1:** Natural product-inspired antifungal drug classes represented by therapeutic entities introduced for human use, with the number and classification of constituent agents and their principal modes of action (MoA) and primary biological processes.

Section No. Class (×Number of Agents)	No. of Agents ^1,2^	NP	NP-D	Principal Mode(s) of Action (MoA)	Primary Biological Process(es)
3. Benzoic acid and salicylic acids (×8)	2 NP 6 NP-D	2	6	Salicylanilide: mitochondrial membrane disruption and antivirulence Salicylaldehyde: covalent Schiff base and metal chelation	Multiple
4. Polyenes (×9)	4 NP complexes 4 NP 1 NP-D complex	8	1	Ergosterol binding, membrane perturbation and pore formation	Cell membrane
5. Griseofulvin (×1)	1 NP	1	–	Disruption of microtubule dynamics and spindle function	Mitosis
6. Pyrithiones (×2)	2 NP-D	–	2	Metal ionophore activity, copper stress, iron starvation, iron–sulfur-cluster damage and disruption of membrane potential and metal homeostasis	Multiple
7. Echinocandins (×4)	4 NP-D	–	4	β-1,3-D-glucan synthase inhibition	Cell wall
8. Fungerp (×1)	1 NP-D	–	1	β-1,3-D-glucan synthase inhibition	Cell wall
9. Fatty acids (×4)	3 NP 1 NP-D	3	1	Antivirulence activity, oxidative-stress induction and inhibition of fatty acid and ergosterol biosynthesis	Multiple
10. Antimetabolite (×1)	1 NP-D	–	1	RNA disruption and thymidylate synthase inhibition	RNA/DNA synthesis
Total	30	14	16		

^1^ Some early antifungal agents entered human therapeutic use before formal regulatory approval systems were established or consistently applied. ^2^ Counts refer to therapeutic entities, not to individual congeners of marketed complexes, salt forms, formulations, trade names or fixed-dose combination products.

## Data Availability

The dataset compiled and analysed for this review, including the identity, NP origin classification, year and jurisdiction of first introduction or approval, antifungal spectrum, administration route and current usage status of the included agents, is presented in the article and Supplementary Materials.

## Supplementary Materials

The following supporting information can be downloaded at https://www.mdpi.com/article/10.3390/antibiotics15090938/s1. File S1: Excel workbook containing a table of the 30 NP-inspired antifungal drugs and drug complexes included in this review, together with the underlying data used to prepare Figure 13.

## Abbreviations

The following abbreviations are used in this manuscript:

A	aerosol suspension
ABC	ATP-binding cassette
ABCD	amphotericin B colloidal dispersion
ABLC	amphotericin B lipid complex
Asp	*Aspergillus*
BPH	benign prostatic hyperplasia
C	current
Can	*Candida*
CPPS	chronic pelvic pain syndrome
cryo-EM	cryogenic electron microscopy
Cry	*Cryptococcus*
D	discontinued
Der	dermatophytes
EMA	European Medicines Agency
ERG	ergosterol-biosynthesis gene(s)
EU	European Union
FKS	fungal β-1,3-D-glucan synthase catalytic-subunit gene(s)
Fus	*Fusarium*
INN	International Nonproprietary Name
L	limited
L-AMB	liposomal amphotericin B
Mal	*Malassezia*
MoA	mode of action
MRSA	methicillin-resistant *Staphylococcus aureus*
NDA	New Drug Application
NP	natural product
NP-D	natural product-derived
O	oral
OTC	over-the-counter
PJP	*Pneumocystis jirovecii* pneumonia
RVVC	recurrent vulvovaginal candidiasis
SAR	structure–activity relationship(s)
T	topical
UK	United Kingdom
US FDA	United States Food and Drug Administration
USA	United States of America
*w*/*w*	weight/weight
